# Pheno- and genotypic iron acquisition of non-*aureus* staphylococci: a scoping review of literature with a focus on bovine mastitis

**DOI:** 10.1186/s13567-025-01613-w

**Published:** 2025-09-25

**Authors:** Helena Reydams, Bruno Toledo-Silva, Nick Vereecke, Freddy Haesebrouck, Sarne De Vliegher

**Affiliations:** 1 M-Team and Mastitis and Milk Quality Research Unit, Department of Internal Medicine, Reproduction, and Population Medicine, Faculty of Veterinary Medicine, Ghent University, 9820 Merelbeke, Belgium; 2 Department of Translational Physiology, Infectiology and Public Health, Faculty of Veterinary Medicine, Ghent University, 9820 Merelbeke, Belgium; 3 PathoSense BV, Lier, Belgium; 4 Department of Pathobiology, Pharmacology and Zoological Medicine, Faculty of Veterinary Medicine, Ghent University, 9820 Merelbeke, Belgium

**Keywords:** Dairy cows, mastitis, non-*aureus* staphylococci, iron-acquisition, WGS, *Staphylococcus chromogenes*, Siderophores

## Abstract

Staphylococci, a heterogenous group of bacteria, are known for their pathogenic potential in both human and animal hosts. Iron acquisition mechanisms play a pivotal role in their virulence and pathogenesis. While *Staphylococcus aureus* has been extensively studied in this regard, there is a significant gap in understanding iron uptake systems among non-*aureus* staphylococci and the closely related mammaliicocci (NASM), particularly those associated with bovine mastitis. This review delves into the diverse strategies employed by staphylococci to scavenge iron from host sources, encompassing extraction from ferritin, heme, transferrin, and lactoferrin. Furthermore, the dearth of knowledge regarding iron acquisition from the host in NASM, with a focus on bovine mastitis, is highlighted. A scoping review of previously published phenotypic and genotypic data highlights key findings on iron uptake in bovine-associated NASM and presents novel genomic findings, in which selected NASM isolates are screened for the first time for xenosiderophore systems, inorganic iron uptake systems, and putative ferritin iron acquisition genes. These results contribute to a more comprehensive understanding of NASM–host interactions. Such insights can pave the way for alternative therapeutic strategies, including leveraging the potential protective effects of specific NASM species or strains against major mastitis pathogens.

**Supplementary Information:**

The online version contains supplementary material available at 10.1186/s13567-025-01613-w.

## Introduction

The genus *Staphylococcus* comprises a diverse group of facultative aerobic Gram-positive bacteria, with over 70 species [1]. Historically, staphylococci have been primarily classified as coagulase-positive or -negative based on their ability to clot rabbit plasma (coagulase tests) with *Staphylococcus aureus* (*S. aureus*) representing the most notable and pathogenic coagulase-positive (CPS) member of the genus [2]. In contrast, the coagulase-negative staphylococci (CNS) were initially considered to be nonpathogenic commensals of the skin and mucous membrane or dismissed as culture contaminants in humans and animals. Since the 1970s and 1980s, however, various CNS species have been increasingly recognized as minor or opportunistic pathogens in both humans and animals and are known to inhabit diverse ecological niches, including environmental sources. Some CNS species (e.g. *S. epidermidis* and *S. haemolyticus*) have become significant nosocomial (hospital-acquired) pathogens in humans [3–7]. Despite nomenclature changes complicating historical interpretations, the pathogenicity of CNS clearly varies considerably among species [3]. Furthermore, exchange of human- and animal-associated CNS between humans, livestock, animal food products, and companion animals, should be considered, underscoring their ecological versatility and importance in public health considerations [4].

An alternative classification scheme to distinguish all staphylococci from *S. aureus* into a single category is “non-*aureus* staphylococci” (NAS), which has been adopted in more recent bovine mastitis literature [5]. This is mainly because some animal (bovine)-associated staphylococcal species display variable ability to coagulate plasma in the coagulase test (e.g., *S. hyicus*, *S. agnetis*, and *S. chromogenes*) [4, 6, 7] and bovine-associated coagulase-negative variants of *S. aureus* have been described [8]. Some human-associated NAS species such as *S. intermedius*, *S. delphini*, *S. lutrae*, *S. pseudointermedius*, and *S. schleiferi* subsp. *coagulans* are coagulase positive [4]. Additionally, based on a recent phylogenomic analysis performed by Madhaiyan et al. [9], five staphylococcal species (*S. sciuri*, *S. fleurettii*, *S. lentus*, *S. stepanovicii*, and *S. vitulinus*) were reassigned to the novel genus *Mammaliicoccus*.

The CNS species have been implicated in foreign body-related infections (e.g., catheter-related), blood stream infections, and urinary tract infections in both humans and animals, as reviewed by Becker et al. [4]. They are also therapeutically challenging for both human and animal health due to the presence of (mobile) antimicrobial resistance mediators [4, 10]. Although virulence factors associated with staphylococcal pathogenicity are not as clearly established for CNS in humans (and to an even smaller extent in animals) as they are in *S. aureus*, a wide range of genes encoding various virulence factors have been identified in different (bovine-associated) staphylococcal species. These factors include those involved in biofilm formation, exotoxins, exoenzymes, host immune invasion factors, and traits related to iron uptake have been identified in different (bovine-associated) staphylococcal species [4, 11]. Collectively, describing specific factors involved in CNS pathogenesis is of vital importance for both human and animal health.

Non-*aureus* staphylococci and the closely related mammaliicocci (NASM) are increasingly being recognized as etiological agents of several infections in animals, including intramammary infections in dairy cows, resulting in an inflammation of the mammary gland (mastitis) [12]. Bovine mastitis is an important and detrimental disease in the dairy industry associated with loss of milk production and quality [13] and high antimicrobial usage for therapeutic and preventative treatment [14, 15].

In the context of bovine mastitis, *S. aureus* is considered to be a major pathogen known for its ability to cause severe clinical mastitis (i.e., visible local or systemic signs) but more often chronic subclinical mastitis (i.e., no visible signs) [16]. Non-*aureus* staphylococci and the closely related mammaliicocci are generally considered to be minor (opportunistic) pathogens with species-specific and on average mild effects for udder health and absent or even positive effects on milk production [5, 17, 18]. At least 25 different NASM species have been isolated from bovine milk samples so far*,* with five predominant species, namely *S. chromogenes, S. epidermidis*, *S. haemolyticus*, *S. simulans*, and *S. xylosus* [16]. Of note, some strains of *S. haemolyticus* originating from human skin and blood with considerable phenotypic and genotypic variation, were reclassified to the novel species *S. borealis* [19]. This species, which was recently isolated from bovine milk in a Turkish study [20], has also been identified in milk samples in the M-team lab in Belgium (non-published data). In most studies, NASM are reported to be the most frequently isolated bacteria from udder quarters with subclinical mastitis [21–24] in dairy herds implementing diverse mastitis control programs and preventative measures (e.g., 10 point plan of the National Mastitis Council [25]). Additionally, NASM can cause mild clinical mastitis cases [5]. In vivo evidence that NASM challenge could modulate local mammary gland immunity exists [26] potentially explaining protective effects against (clinical) mastitis and even (indirect) positive effects for milk yield.

Although iron acquisition in Gram-positive bacteria, including *S. aureus*, has been extensively reviewed [26–35], a comprehensive overview of this topic is lacking for staphylococci other than *S. aureus*. Throughout this review, all staphylococci other than *S. aureus* will be referred to as NASM for both human, animal, and bovine-associated species rather than using CPS and CNS when referring to human/animal-associated species and when referring specifically to bovine-associated species. In addition to reviewing the available literature, new data of putative iron acquisition proteins based on whole genome sequencing (WGS) (Table 1) are presented.

**Table 1:** **Putative genetic loci, other than heme- and siderophore-related, involved in iron acquisition for bovine-associated non-*aureus* staphylococci and mammaliicocci species isolated in Canada (*n*** **= 441) [**11**] and Belgium (*n* = 9) [**41, 46]

Bovine NASM	Xenosiderophores									Inorganic iron						Putative ferritin iron acquisition		
	Ferric hydroxamate uptake					Sst catechol transporter				Ferrous iron ABC transporter			Staphylococcal ferrous iron transporter					
Canada	fhuB	fhuG	fhuC	fhuD1	fhuD2	sstA	sstB	sstC	sstD	feoA	feoB	feoC	sitA	sitB	sitC	Oxidoreductase	Monooxygenase	Probable membrane protein
S. AGN (13)	100	100	100	38	100	100	100	100	100	100	100	0	100	100	77	100	0	0
S. ARL (15)	93	100	100	0	100	100	100	100	100	0	0	0	100	100	100	100	100	100
S. AUR (2)	100	100	100	100	100	0	0	100	0	0	0	0	100	100	100	100	100	100
S. BOR (2)	100	100	100	0	100	100	100	100	100	0	0	0	100	100	100	100	0	100
S. CAPI (22)	100	100	100	0	100	0	0	100	0	100	100	0	100	100	100	100	0	0
S. CAPR (1)	0	100	100	0	100	100	100	100	100	100	100	0	100	100	0	100	100	0
S. CHR (83)	100	100	100	0	100	100	100	100	100	100	100	0	100	100	95	100	0	0
S. COH (24)	100	100	100	0	100	100	96	100	100	100	100	0	100	100	100	100	100	100
S. DEV (8)	75	100	100	100	100	100	100	100	100	0	0	0	100	100	100	100	100	0
S. EPI (26)	0	100	100	0	0	100	100	100	92	100	100	0	100	100	100	100	100	0
S. EQU (17)	100	100	100	12	100	100	100	100	100	100	100	0	100	100	94	100	100	100
M. FLE (1)	100	100	100	100	100	100	100	100	100	0	0	0	100	100	100	100	100	100
S. GAL (21)	100	100	100	100	100	100	100	100	100	95	95	0	100	100	100	100	100	100
S. HAE (29)	100	100	100	0	100	100	100	100	100	0	0	0	100	100	100	100	14	100
S. HOM (11)	93	100	100	0	0	100	100	100	100	73	100	0	100	100	73	100	100	100
S. HYI (3)	0	100	100	100	100	100	100	100	100	100	100	0	100	100	100	100	0	0
S. KLO (1)	100	100	100	0	100	100	100	100	100	0	0	0	100	100	100	100	100	100
S. NEP (2)	100	100	100	0	100	100	100	100	100	100	100	0	100	100	100	100	100	100
S. PAS (6)	100	100	100	0	100	100	100	100	100	0	0	0	100	100	100	0	0	0
S. SAP (16)	67	100	100	6	100	100	100	100	100	100	6	0	100	100	63	100	100	100
M. SCI (29)	100	100	100	86	100	100	100	100	100	0	0	0	0	0	0	0	0	0
S. SIM (42)	100	100	100	0	100	100	100	100	100	100	100	0	100	100	100	100	100	100
S. SUC (15)	100	100	100	0	100	100	100	100	100	0	0	0	100	100	100	100	100	100
M. VIT (6)	87	100	100	0	100	100	100	100	100	0	0	0	100	100	0	100	100	100
S. WAR (19)	100	100	100	26	100	100	100	100	100	100	100	0	100	100	100	100	37	100
S. XYL (28)	100	100	100	21	100	100	100	100	100	46	46	0	100	100	100	96	100	100
Belgium																		
S. CHR (4)	100	100	100	0	100	100	100	100	100	100	100	0	100	100	100	100	0	0
S. EQU (2)	100	100	100	0	100	100	100	100	100	100	100	0	100	100	100	100	100	100
S. SIM (3)	100	100	100	0	100	100	100	100	100	100	100	0	100	100	100	100	100	100

Values within cells represent the percentage of isolates containing the given gene. Species abbreviations: S. AGN, *S. agnetis*; S. ARL, *S. arlettae*; S. AUR, *S. auricularis*; S. BOR, *S. borealis*; S. CAPI, *S. capitis*; S. CAPR, *S. caprae*; S. CHR, *S. chromogenes*; S. COH, *S. cohnii*; S. DEV, *S. devriesei*; S. EPI, *S. epidermidis*; S. EQU, *S. equorum*; M. FLE, *M. fleurettii*; S. GAL, *S. gallinarum*; S. HAE, *S. haemolyticus*; S. HOM, *S. hominis*; S. HYI, *S. hyicus*; S. KLO, *S. kloosii*; S. NEP, *S. nepalensis*; S. PAS. *S. pasteuri*; S. SAP, *S. saprophyticus*; M. SCI, *M. sciuri*; S. SIM, *S. simulans*; S. SUC, *S. succinus*; M. VIT. *M. vitulinus*; S. WAR, *S. warneri*; and S. XYL, *S. xylosus*.

## Materials and methods

This review summarizes the numerous iron acquisition mechanisms currently identified in staphylococci, along with the known genes underlying the iron uptake systems in bovine mastitis-associated NASM. Since our current understanding on staphylococcal iron systems originates primarily from studying mechanisms in *S. aureus*, it is used as reference. Studies on the genes associated with iron acquisition in (bovine-associated) NASM and their preferred iron uptake systems in specific tissue sites are limited, particularly phenotypic testing combined with genotypes for bovine-associated NASM. To our knowledge, only three studies have assessed the phenotypic growth characteristics of bovine-associated NASM in growth media with different concentrations of iron [36–38] and 5 studies have reported iron uptake genes in a number of bovine (mastitis)-associated NASM [11, 16, 36, 39, 40] through WGS analyses (Additional file 1 and Table 2).

**Table 2:** **Genetic loci involved in heme- and siderophore-related iron acquisition in bovine-associated non-*aureus* staphylococci and mammaliicocci**

Bovine NASM	Heme (isd)									Endogenous Siderophores																		
										Staphyloferrin A							Staphyloferrin B											
	Binding and extraction			Import				Degredation		Synthesis and export				Import and release			Synthesis and export									Import and release		
	isdA	isdB	isdH	isdC	isdD	isdE	isdF	isdG	isdI	sfaA	sfaB	sfaC	sfaD	htsA	htsB	htsC	sbnA	sbnB	sbnC	sbnD	sbnE	sbnF	sbnG	sbnH	sbnI	sirA	sirB	sirC
S. AGN (17)	0	0	0	0	0	0	0	0	100	100	100	100	100	100	100	100	100	100	100	100	100	100	100	100	100	100	100	100
S. ARL (16)	0	0	0	0	0	0	6	94	6	0	100	100	100	6	6	100	100	94	94	94	94	94	94	94	94	88	100	100
S. AUR (3)	0	0	0	67	0	67	100	33	100	100	100	67	100	100	100	100	100	0	0	0	0	0	0	0	0	33	33	0
S. BOR (2)	0	0	0	0	0	0	0	0	0	100	100	100	100	100	100	100	0	0	0	0	0	0	0	0	0	0	0	0
S. CAPI (22)	95	0	0	100	0	100	100	100	100	100	100	95	100	100	100	100	100	0	0	0	0	0	0	0	0	0	0	95
S. CAPR (1)	0	0	0	100	0	100	100	100	100	100	100	100	100	100	100	100	100	0	0	0	0	0	0	0	0	0	100	100
S. CHR (206)	1	0	0	0	0	0	0	3	49	100	100	99	100	99	100	100	98	1	0	0	0	0	0	0	0	7	100	98
S. COH (26)	0	0	0	0	0	0	42	4	100	100	92	92	100	100	100	100	100	46	0	0	4	0	0	0	0	8	8	62
S. DEV (10)	0	0	0	0	0	0	20	20	100	90	100	100	100	100	100	100	100	0	0	0	0	0	0	0	0	100	100	100
S. EPI (33)	0	0	0	0	0	0	21	100	18	100	100	100	100	100	100	100	100	0	0	0	0	0	0	0	0	0	67	3
S. EQU (21)	0	0	0	0	0	0	10	10	90	0	100	100	100	100	100	100	100	100	95	100	95	95	95	95	95	100	100	100
M. FLE (2)	0	0	0	0	0	0	100	0	100	0	100	0	0	0	100	0	100	0	0	0	0	0	0	100	0	100	0	100
S. GAL (21)	0	0	0	0	0	0	5	0	100	100	100	100	95	100	100	100	100	0	0	0	0	0	0	0	0	57	5	0
S. HAE (38)	0	0	0	0	0	0	21	0	74	100	100	82	100	95	100	100	100	0	0	0	0	0	0	0	0	68	95	100
S. HOM (15)	0	0	0	0	0	0	33	27	100	0	100	100	93	80	100	100	100	0	0	0	0	0	0	0	0	0	0	0
S. HYI (4)	0	0	0	0	0	0	25	25	100	100	100	100	100	100	100	100	100	100	100	100	100	100	100	100	100	100	100	100
S. KLO (1)	0	0	0	0	0	0	0	0	100	100	100	100	100	100	100	100	100	0	0	0	0	0	0	0	0	100	0	0
S. NEP (2)	0	0	0	0	0	0	0	0	100	100	100	100	100	100	100	100	100	0	0	0	0	0	0	0	0	100	100	0
S. PAS (6)	33	0	100	100	0	100	100	100	83	100	100	100	100	100	100	100	100	0	0	0	0	0	0	0	0	0	0	100
S. SAP (17)	0	0	0	0	0	0	0	6	100	100	100	100	100	100	100	100	100	0	0	0	0	0	0	0	0	94	6	6
M. SCI (34)	0	0	0	50	0	50	100	0	53	0	0	0	0	88	91	100	100	0	0	0	0	0	9	44	0	91	6	44
S. SIM (173)	3	5	68	30	68	68	31	6	99	100	100	100	100	87	100	100	98	0	0	0	0	0	0	0	0	0	3	3
S. SUC (15)	0	0	0	0	0	0	0	100	0	100	100	100	100	100	100	100	100	0	0	0	0	0	0	0	0	0	93	0
M. VIT (7)	0	0	0	0	0	0	100	0	100	86	0	0	0	14	100	100	100	0	0	0	0	0	0	100	0	100	0	100
S. WAR (21)	0	0	0	0	0	0	81	10	100	100	100	100	100	100	100	100	100	0	0	0	0	0	0	0	0	10	10	29
S.XYL (30)	0	0	0	0	0	0	7	7	100	100	97	100	100	100	100	100	100	0	0	0	0	0	0	0	0	97	63	43

The same non-*aureus* staphylococcus and Mammaliicoccus species across different studies are grouped together. Values within cells represent the percentage of isolates containing the given gene. Species abbreviations: S. AGN, *S. agnetis*; S. ARL*, S. arlettae*; S. AUR, *S. auricularis*; S. BOR, *S. borealis*; S. CAPI, *S. capitis*; S. CAPR, *S. caprae*; S. CHR, *S. chromogenes*; S. COH, *S. cohnii*; S. DEV, *S. devriesei*; S. EPI, *S. epidermidis*; S. EQU, *S. equorum*; M. FLE, *M. fleurettii*; S. GAL, *S. gallinarum*; S. HAE, *S. haemolyticus*; S. HOM, *S. hominis*; S. HYI, *S. hyicus*; S. KLO, *S. kloosii*; S. NEP, *S. nepalensis*; S. PAS. S. *pasteuri*; S. SAP, *S. saprophyticus*; M. SCI, *M. sciuri*; S. SIM, *S. simulans*; S. SUC, *S. succinus*; M. VIT. *M. vitulinus*; S. WAR, *S. warneri*; and S. XYL, *S. xylosus*. The isolates originate from from Finland (*n* = 20) (Åvall-Jääskeläinen et al. [16]), Canada (*n* = 441) (Naushad et al. [11]), Belgium and Norway (*n* = 50) (Fergestad et al. [39]), Sweden (*n* = 223) (Waller et al. [40]), and Belgium (*n* = 9) (the M-team repository [41] and Reydams et al. [36]).

### Search strategy and selection criteria

The literature search targeted full-length research articles and review papers published between 1970 and 2024. Electronic databases searched included PubMed, ScienceDirect, Scopus, Springer Link, Wiley Online Library, and Google Scholar. A structured search strategy was employed using Boolean operators (“AND”, “OR”) to refine results. Key search terms included bovine, mastitis, coagulase-negative staphylococci, non-aureus staphylococci, iron acquisition, iron uptake, siderophores, heme, ferritin, lactoferrin, Fur, xenosiderophores, virulence factors, antimicrobial resistance, genotypic identification, phenotypic identification, and whole genome sequencing. Searches were performed within the title, abstract, and keyword fields of publications.

A chain-based search approach was adopted, beginning broadly with general concepts such as bovine mastitis and iron acquisition, then progressively focusing on specific aspects including NASM virulence factors, phenotypic iron acquisition traits, and putative genes linked to iron uptake. Relevant secondary studies were identified through citation tracking. Studies were included if they provided data on NASM isolates associated with mastitis in bovine or other domestic ruminants, or if they described phenotypic or genotypic characterization, iron acquisition systems, virulence, antimicrobial resistance, or biofilm mechanisms. Studies focusing exclusively on *S. aureus* without comparison to NASM, non-peer-reviewed articles, editorials, conference abstracts, and studies unrelated to iron metabolism or staphylococcal pathogenicity were excluded. All identified records underwent initial screening based on titles and abstracts, followed by a comprehensive full-text review. Relevant publications were logged systematically, extracting data on pathogen species, source, host, virulence factors, and iron-related genetic findings.

### Whole genomic sequencing and phylogenetic analysis

The whole genome sequencing and phylogenetic analysis was conducted as described by Reydams et al. [36]. Briefly, complete genome sequences from bovine-associated NASM from a study by Åvall-Jääskeläinen et al. [16] (*n* = 20), Naushad et al. [11] (*n* = 441), Fergestad et al. [39] (*n* = 50), Waller et al. [40] (*n* = 223), from the M-team repository [41] (*n* = 3) and from Reydams et al. [36] (n = 6) were screened for siderophore and putative iron-uptake associated genes using a custom protein database (see Additional file 2) in Abricate (v.1.0.1) [70] with minimal query coverage and amino acid homology set to 30 and 50%, respectively. The first genomic hits that met the minimum cutoff for each individual query were selected. Trees and identified proteins were visualized in iTOL (v.5) [61]. For the data analyses of short-read data, the raw read files from the above-mentioned studies [11, 16, 39, 40] were downloaded from NCBI and subjected to assembly using default pipelines as described their respective manuscripts to not deviate from their original data.

## Mammalian host iron metabolism and storage

Iron is an essential trace element that serves as an enzymatic co-factor in various biological processes, including electron transfer through oxidation–reduction reactions, oxygen transport, oxidative phosphorylation, DNA biosynthesis, and xenobiotic (foreign compounds) metabolism [42]. Due to its divalent nature, iron alternates between its oxidized ferric (Fe^3+^; aerobic environment) and reduced ferrous (Fe^2+^; acidic/anaerobic environment) states. Ferrous iron easily undergoes the Fenton reaction with hydrogen peroxide, consequently creating hydroxyl radicals which can cause peroxidation of lipid membranes, oxidative damage of DNA, and other cytotoxic effects [43–46]. To prevent these deleterious effects, iron is complexed with other molecules or proteins to keep it in a soluble, bio-available, non-toxic form [47, 48].

Iron reservoirs in the body are ferritin, heme, heme carrier proteins, transferrin, and lactoferrin. Surplus of iron in the host is stored intracellularly by ferritin, an iron-binding ubiquitous protein largely responsible for iron homeostasis. Ferritin stores iron in a bioavailable form while keeping it in a non-toxic state [45, 49]. Most of the intracellular iron pool, however, is found within heme and heme carrier proteins [28]. Heme is a molecular iron-containing complex (a planar tetrapyrrole ring with a central ferrous iron ion) bound to metalloproteins such as the hemoglobin in erythrocytes or myoglobin in skeletal muscles [34, 50]. In case of erythrocyte lysis (biological or pathological), hemoglobin is released into the extracellular compartment and undergoes oxidative degradation, releasing reactive heme and its toxic by-product ferrous iron into the serum [51–54]. Free hemoglobin is quickly scavenged and irreversibly bound by the glycoprotein haptoglobin, forming a soluble complex that is specifically recognized by receptors on macrophages and hepatocytes for its readily degradation [55, 56]. Free serum heme is scavenged and delivered to hepatocytes by hemopexin, a high affinity heme-binding protein, while ferrous iron is bound by calprotectin [32, 35].

Transferrin, an extracellular glycoprotein that binds free ferric iron in serum, is mainly responsible for iron distribution throughout the body by complexing and donating iron to recipient iron-dependent cells via one or multiple transferrin cell membrane receptors [33]. Lactoferrin, likely an analogue of serum transferrin, is a bacteriostatic glycoprotein sequestering and transporting free ferric iron in secretions such as milk, saliva, tears, and mucosal surfaces [57, 58]. During an infection, lactoferrin is also released by polymorphonuclear leukocytes, increasing its concentration in bodily secretions [59].

### Staphylococcal iron acquisition

The success of staphylococci as pathogens has been attributed, in part, to their ability to acquire iron from the host. Upon host recognition of pathogens in a mammalian host, a non-specific host defense mechanism (also referred to as nutritional immunity) is activated whereby intracellular and extracellular iron availability is limited below the threshold supporting bacterial proliferation and survival [48, 60]. Most microorganisms have developed high-affinity iron transport systems to counteract the host's nutritional immunity, which withholds free iron necessary for bacterial growth. These systems import iron primarily as a substrate bound to a ligand [51, 61].

Staphylococci have developed multiple components to acquire, transport, and store iron from the extracellular compartment into the bacterial cytoplasm by utilizing an integral cytoplasmic membrane protein, an ATP-binding-cassette (ABC) transporter, to actively transport iron across the cell membrane. The transporter consists of three domains; first a periplasmic binding protein that recognizes the ligand bound to its iron substrate, second a transmembrane permease that permits transport through the lipid bilayer of the cytoplasmic membrane, and third an ATPase, that hydrolyses ATP to drive translocation of the iron substrate into the cytoplasm [62]. In comparison to *S. aureus*, information regarding the iron acquisition mechanisms for (bovine-associated) NASM is scarce (Table 3) yet takes on added significance when the pathogenic and/or protective potential of different NASM species or strains is considered [26, 63, 64].

**Table 3:** **Publications studying iron acquisition in human and animal-associated non-*aureus*** **staphylococcal and mammaliicoccal (NASM) species**

NASM species	Origin	References
*S. hyicus*	Pig	Konetschny-Rapp et al. [113]
*S. hyicus*	Pig	Meiwes et al. [114]
*S. epidermidis*, *S. hominis*, *S. warneri*, *S. simulans*, *S. capitis, S. haemolyticus*, *S. saprophyticus*	Human	Wilcox et al. [115]
*S. epidermidis*	Human	Modun et al. [116]
*S. hyicus*	Pig	Drechsel et al. [117]
*S. hyicus*	Pig	Haag et al. [118]
*S. epidermidis,* S. *haemolyticus, S. warneri, S. caprae*	Human (clinical isolates)	Lindsay and Riley [119]
*S. epidermidis*	Human	Lindsay et al. [90]
*S. epidermidis*	Human	Heidrich et al. [120]
*S. epidermidis*, *S. hominis*, *S. cohnii*, *S. lugdunensis*, *S. warneri*, *S. haemolyticus*	Human (clinical isolates)	Cockayne et al. [121]
*S. epidermidis, S. cohnii*, *S. hominis, S. lugdunensis*, *S. haemolyticus*	Human	Hill et al. [102]
*S. carnosus*	Dry sausage	Hill et al. [102]
*S. epidermidis, S. saprophyticus*	Human	Modun et al. [91]
*S. epidermidis*	Human	Modun et al. [92]
*S. epidermidis*	Human	Freestone et al. [97]
*S. epidermidis, S. haemolyticus, S. warneri,* S. cohnii, *S. lugdunensis*, *S. saprophyticus*	Human	Morrissey et al. [122]
*S. epidermidis, S. capitis, S. cohnii, S. haemolyticus, S. hominis, S. warneri, S. xylosus*	Human	Neal et al. [98]
*S. epidermidis*	Human	Lyte et al. [99]
*S. auricularis, S. capitis*, *S. caprae*, *S. chromogenes, S. cohnii, S. epidermidis, S. haemolyticus, S. intermedius, S. hominis, S. sciuri, S. simulans, S. warneri*, *S. xylosus*	Human, goat (milk), pig, squirrel	Dale et al. [81]
*S. epidermidis*	Human	Massonet et al. [103]
*S. epidermidis*	Human	Beasley et al. [78]
*S. capitis, S. epidermidis*, *S. saprophyticus*, *S. haemolyticus*, *S. carnosus*, *S. hominis*	Human, dry sausage	Beasley and Heinrichs [67]
*S. lugdunensis*	Human (breast abscess)	Haley et al. [75]
*S. lugdunensis*	Human (breast abscess)	Heilbronner et al. [123]
*S. pseudintermedius, S. delphini, S. intermedius*, *S. epidermidis*, *S. haemolyticus*, *S. saprophyticus*, *S. carnosus*	Dog, horse, bird, human, dry sausage	Ben Zakour et al. [83]
*S. epidermidis, S. lugdunensis, S. saprophyticus, S. haemolyticus, S. pseudintermedius, S. warneri, S. capitis, S. caprae, S. hominis, S. carnosus, and S. xylosus*	not specified	Sheldon and Heinrichs [2]
*S. lugdunensis*	Human (breast abscess, endocarditis)	Zapotoczna et al. [76]
*S. lugdunensis*	Human skin	Brozyna et al. [85]
*S. arlettae, S. equorum*	not specified	Kobylarz et al. [84]
*25 NASM species*	Cow: (Sub)clinical mastitis	Naushad et al. [11]
*S. saprophyticus*	Human	Souza et al. [124]
*S. pseudintermedius*	Dog	Verstraete et al. [125]
*S. chromogenes, S. cohnii*, *S. equorum, M. fleurettii, S. haemolyticus*, and *S. rostri*	Cow: (Sub)clinical mastitis, rectal feces, and teat apex	Wuytack et al. [38]
*S. epidermidis, S. caprae, S. capitis*	Human, Buffalo (milk), **Cow**, Goat (milk), Kefir	Sun et al. [77]
*16 NASM species*	Cow: (Sub)clinical mastitis	Fergestad et al. [39]
*S. epidermidis*	Human	Oliveira et al. [89]
*S. lugdunensis*	not specified	Flannagan et al. [63]
*S. chromogenes and S. simulans*	Cow: subclinical mastitis	Waller et al. [40]

**Figure 1:**
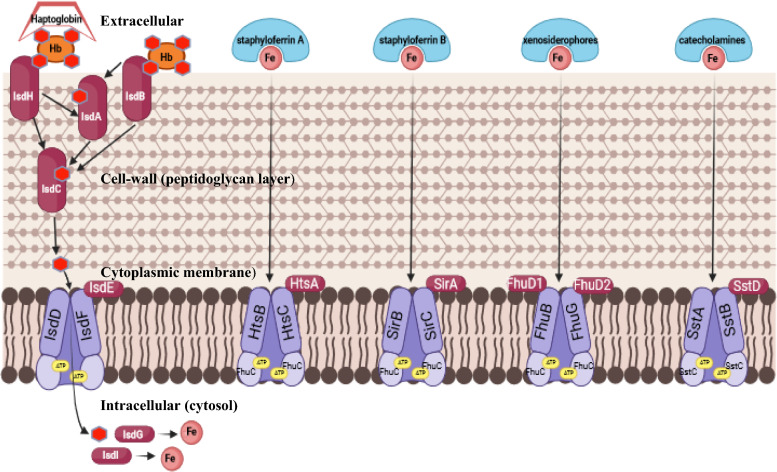
**Iron acquisition systems in *Staphylococcus aureus*** **and for some (bovine-associated) non-*aureus*** **staphylococci and mammaliicocci.** Heme can be acquired from hemoglobin (Hb) and haptoglobin-Hb complexes using the Isd system. Cell-wall anchored proteins IsdH, IsdB and, IsdA acquire heme from the extracellular compartment, which is transferred via IsdC to the cytoplasmic membrane IsdEF transporter. In the cytosol iron (Fe) is released from heme. Additionally, various siderophores like staphyloferrin A and B, xenosiderophores, and catecholamines can be taken up via HtsABC, SirABC, FhuBGCD_1_D_2_, and SstABCD ABC cytoplasmic membrane transporters, respectively. This figure was adapted from Adolf [112] and created using Biorender.com.

So far, the iron-uptake mechanisms identified in staphylococci includes (1) iron extraction from ferritin, (2) the uptake of heme–iron, (3) the production and secretion of high-affinity iron-scavenging siderophores to acquire iron from transferrin and lactoferrin, (4) receptors for xenosiderophore uptake, and (5) the uptake of free ferrous iron, as reviewed by Sheldon et al. [34] (Figure 1).

#### Iron uptake regulation by Fur

A decrease in intracellular iron is a vital sensory trigger for the activation of iron uptake pathways in bacteria. For Gram-positive *Firmicutes*, such as *Staphylococcus* spp., iron transport system gene transcription is regulated by the ferric uptake regulation (Fur) metalloprotein, a transcriptional repressor first discovered in *Escherichia coli* [65]. Whenever there is enough iron in the extracellular medium, the Fur protein represses transcription of the iron-regulated uptake genes and biosynthesis of siderophores [66]. When iron is readily available, ferric iron binds to each Fur monomer causing a conformational change to the dimeric protein facilitating its binding to its target DNA sequence, known as the Fur box. This Fur box lies within the promoter region of iron-regulated genes and acts as a repressor for the transcription of almost all genes and operons required for iron-uptake. When iron is limited in the environment, ferric iron dissociates from the Fur protein, resulting in the release of the Fur repressor and allowing RNA polymerase access to the Fur box. This subsequently results in active transcription and translation of all iron transport systems [65, 67, 68].

#### Iron extraction from ferritin

To our knowledge nothing is known about the mechanism for ferritin iron acquisition by staphylococci. A study by Vermassen et al. [69] found a *S. xylosus*^H (human-associated)^ strain to grow in the presence of ferritin as a sole iron source in which a three-cistron operon was highly overexpressed under ferritin iron growth conditions revealing the involvement of a potential reductive pathway. This gene cluster was also found to be present in certain NASM species (origin not specified) including, *S. saprophyticus*, *S. equorum*, *S. cohnii*, *S. gallinarum*, and *S. succinus* when performing a BLAST-based screening [69]. Additionally, several *S. hominis*^B (bovine−associated)^ and *S. chromogenes*^B^ isolates presented significant growth recovery in iron-poor media with ferritin as the sole iron source [37]. In a subsequent study, putative ferritin iron acquisition genes were identified in *S. chromogenes*^B^ and *S. equorum*^B^ isolates based on WGS analyses [36]. When performing WGS, this three gene reductive pathway (oxidoreductase, monooxygenase, and a probable membrane protein) was also identified in most bovine-associated NASM, as shown in Table 1, with some strain variation noted for the presence/absence of the monooxygenase gene in *S. haemolyticus* and *S. warneri*.

#### Iron extraction from heme and heme-binding proteins: Isd system

*Staphylococcus aureus* has an *i*ron regulated *s*urface *d*eterminant (*isd*) locus, comprised of nine *isd* genes (named A through I, respectively) involved in iron uptake from hemoglobin and haptoglobin-hemoglobin complex, its preferred iron source [70]. On the contrary, binding of hemopexin to heme inhibits the uptake of heme by the *S. aureus* Isd system [35]. When the Fur repressor protein is removed from its Fur box upstream of the *isd* locus, heme acquisition from heme-containing proteins is mediated by transporting captured heme from the extracellular medium through the cell wall and cytoplasmic membrane where heme–iron can be extracted [28, 34, 47, 67, 70–73]. The Isd system has typically only been described in *S. aureus* but more recent studies identified a homologous system in *S. lugdunensis*^H^, which was considered to be unique to NASM [63, 74–76]*.* A study by Sun et al. [77] however, also identified novel *isd* homologues in strains of *S. caprae*^H, A (animal−associated),B^ and *S. capitis*^H^ and recent studies performing WGS on a select group of bovine-associated NASM isolates identified a number of *isd* genes in some species (e.g., *S. simulans*^B^ and *S. pasteuri*^B^) [11, 16, 39], which suggests their potential to acquire heme. Heme extraction from myoglobin has been shown in *S. aureus* but this process has not been investigated in depth [32].

#### Iron extraction from heme: Fep system

The *F*e-*d*ependent *p*eroxidase (FepABC) system, first identified in *S. aureus*, has been proposed as an alternative heme–iron uptake system by binding heme in analogy to the Isd system, resulting in a reduction of iron, and internalization of ferrous iron. So far, the Fep system has only been found in the most virulent human staphylococcal species, including *S. aureus, S. haemolyticus*^H^, and *S. lugdunensis*^H^ [32].

#### Iron extraction from transferrin and lactoferrin: siderophore systems

**Figure 2:**
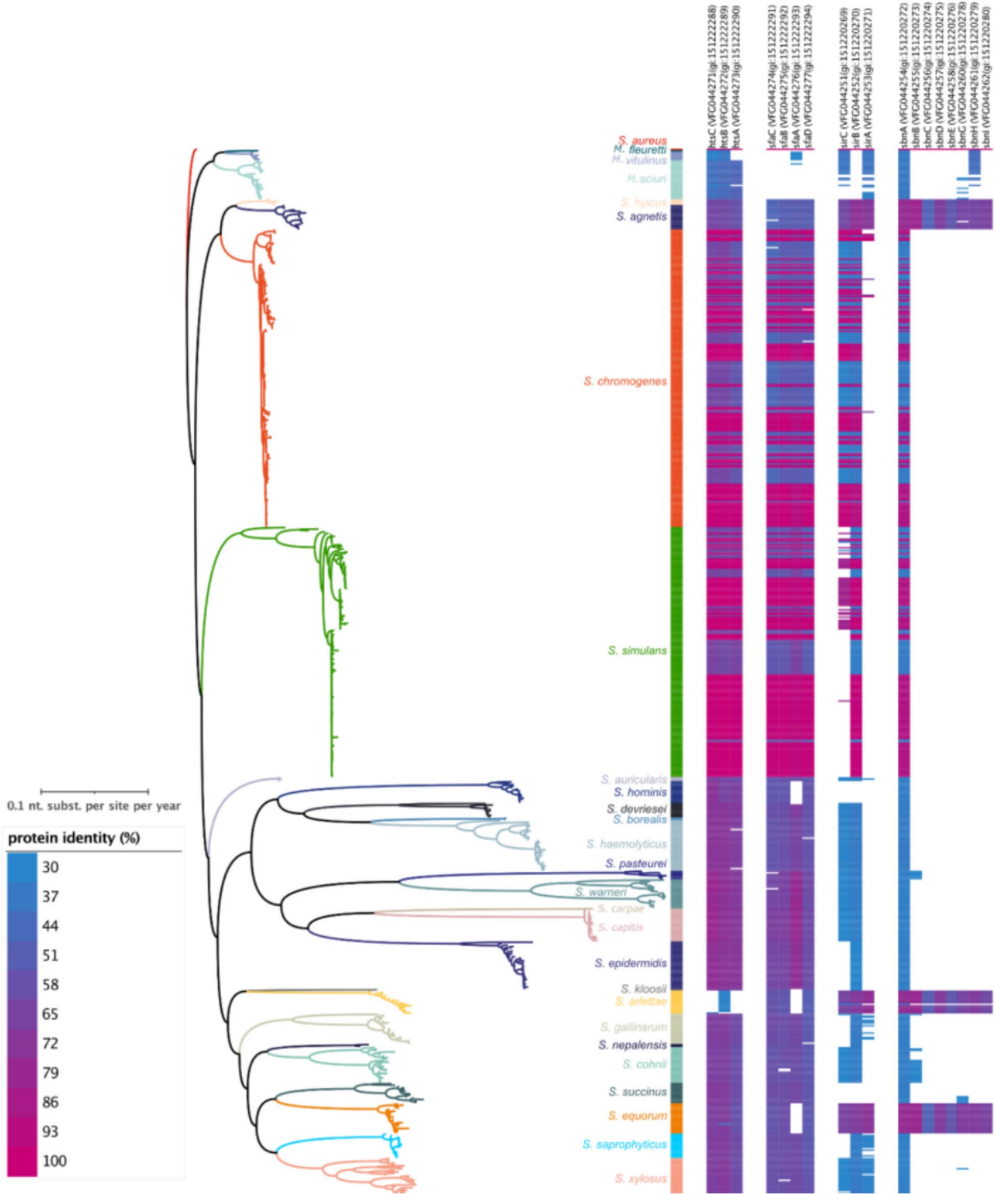
**Presence of siderophore-associated proteins for 26 bovine-associated non-*aureus*** **staphylococci and mammaliicocci species (*n* = 743) using whole genome-sequencing.** The isolates originated from Finland (Åvall-Jääskeläinen et al. [16]), Canada (Naushad et al. [11]), Belgium and Norway (Fergestad et al. [39]), Sweden (Waller et al. [40]), and from Belgium (M-team repository UGent [41] and Reydams et al. [36]) using whole genome sequencing [36].

A common bacterial iron acquisition strategy is the production of siderophores, as low-molecular weight (usually < 1 kDa) ferric iron-chelators that are secreted in response to iron deprivation to scavenge residual free iron from the environment and to expropriate it from host glycoproteins, such as transferrin and lactoferrin [33]. Staphylococci may synthesize and secrete two types of *α*-hydroxycarboxylate type siderophores, staphyloferrin A (SA) and staphyloferrin B (SB). The four-gene *sfa* (staphyloferrin A)-*ABCD* and nine-gene s*bn* (staphyloferrin B)-*ABCDEFGHI* loci, encode biosynthetic proteins responsible for the synthesis of SA [78, 79] and SB [80, 81], respectively (Figure 1). Staphyloferrin A biosynthesis was first discovered in culture supernatants of *S. hyicus*^**A**^ and the *sfa* locus was found to be widely distributed among staphylococci [67]. Staphyloferrin A production relies upon citrate derived from the tricarboxylic acid cycle (TCA) cycle central metabolism. Under iron-restriction the expression of non-essential iron-containing pathways is reduced, including the TCA cycle, in favor of glycolytic and fermentative metabolism; therefore, iron-restriction in glucose-rich media severely hinders SA synthesis and its ability to transport iron into the cells [82]. In contrast to SA, the staphyloferrin B biosynthesis gene cluster (sbn) is mostly limited to more pathogenic staphylococci, such as *S. aureus* and *S. intermedius*^A^ [83]. Though, it is also found in some NASM species, including bovine-associated isolates (*Staphylococcus agnetis*^B^, *S. arlettae*^B^, *S. equorum*^B^, and *S. hyicus*^B^; (Additional file 1 and Table 2 and Figure 2) [11]. Staphyloferrin B is produced from citrate generated by a second citrate synthase (*sbnG*) that is structurally distinct from the TCA cycle citrate synthase [84]. Furthermore the biosynthetic genes for SB are among the most robustly upregulated under iron restriction suggesting that SB synthesis is more conductive for invasive infections while SA is more conductive to promote colonization, as reviewed by Sheldon and Heinrichs [33]. Some staphylococci, such as *S. lugdunensis*^H^, do not synthesize staphyloferrin molecules [63, 85]. Uptake of ferric-SA or ferric-SB is linked to iron-regulated ABC-type transporters HtsABC (heme-*t*ransport *s*ystem; used to be implicated in heme–iron acquisition [78, 86–88] and SirABC (staphylococcal iron-*r*egulated), respectively. The two transport systems are non-interchangeable, meaning they are specific to their respective siderophore [78, 80]. Interestingly, some bovine-associated NASM species, such as *S. devriesei*, *S. chromogenes*, *S. haemolyticus*, *S. warneri*, and *S. xylosus*, showed BLAST hits for the *sirABC* genes across different countries and in varying degrees (Additional file 1 and Table 2), but they lacked most SB synthesis genes. The production and distribution of siderophores SA and SB have been reported to vary in different tissue abscesses, suggesting differential regulation of these two siderophores [27]. Additionally, as observed in *S. epidermidis*^H^, siderophores play a crucial role in biofilm formation by ensuring iron availability [89]. Finally, the presence of a transferrin receptor in *S. aureus* [90] and *S. epidermidis* [91, 92] indicates that these two species can take up iron directly from transferrin but the nature of the transferrin receptor remains controversial in many cases [35].

#### Iron extraction from transferrin and lactoferrin: xenosiderophores

In addition to the production and utilization of endogenous siderophores, staphylococci can appropriate xenosiderophores (i.e., siderophores produced by other staphylococci and microbes) by expressing (1) a matching receptor for uptake (e.g., HtsABC and SirABC) [31] and/or (2) the ferric hydroxamate *u*ptake (Fhu) transporter and the Sst catechol transporter [93, 94]; These are transporters with a broader specificity instead of for a single siderophore. [33]. Exploiting multiple exogenous siderophores without the energetic demands associated with their synthesis and secretion, may provide staphylococci a competitive advantage within heterogenous bacterial populations [33, 63]. *Staphylococcus lugdunensis*^H^, for example, can usurp SA and SB produced by *S. aureus* because HtsABC and SirABC transporters are expressed [63, 85]. Additionally, some strains of *S. chromogenes*^B^ appear to have a complete SirABC transporter locus (Additional file 1 and Table 2), indicating their potential to also import SB. These putative iron acquisition genes are also identified in *S. devriesei*^B^, *S. haemolyticus*^B^, *S. warneri*^B^, and *S. xylosus*^B^ (Additional file 1 and Table 2), suggesting a similar potential for SB-mediated iron acquisition besides SA.

1. Acquisition of hydroxamate-siderophores—Fhu system

Staphylococci are known to use hydroxamate type siderophores, including desferrioxamine B, ferrichrome, aerobactin, and coprogen, as iron sources via the conserved Fhu system (BGCD_1_D_2_) [95] (Figure 1). While FhuD2 functions as the substrate-binding protein with broad specificity, the partially redundant paralog FhuD1 appears to accommodate a more limited range of siderophores [95, 96]. Some staphylococci (e.g., *S. epidermidis* and *S. hominis*^B^) do not possess the FhuD_1_D_2_ uptake system, or they have not been identified so far (Table 1). It is suggested that *fhuC* is needed to support isd-dependent heme acquisition in *S. aureus* and is shown to energize the Hts and Sir uptake systems in *S. aureus* [32]. Additionally, a study by Vermassen et al. [69] identified the ATPase *fhuC* gene in a *S. xylosus*^H^ strain.

2. Acquisition of catecholate xenosiderophores—Sst system

*Staphylococcus* spp., including *S. aureus*, *S. epidermidis*^H^*, S. capitis*^H^*, S. saprophyticus*^H^*, S. haemolyticus*^H^, and *S. hominis*^H^, are stress-hormone responsive which means they are able to use exogenous catecholate siderophores and ferrated catecholamine stress hormones (i.e., epinephrine, norepinephrine, and dopamine) as “pseudosiderophores” via the *sstABCD* operon [97–99] (Table 1). Catecholamines mediate the removal of iron from host transferrin and lactoferrin by reducing ferric iron bound to transferrin to ferrous iron liberating ferrous iron from its protein-complexed form and enabling capture by the hormone. The iron-binding capability of transferrin and lactoferrin is also reduced which allows bacterial pathogens to easily scavenge ferric iron from its host iron-chelator form [60, 100]. The functionality of this locus is only evident when endogenous biosynthesis of SA and SB is disrupted [94]. Evidence suggests that the SstABCD system may have specific roles in staphylococcal pathogenesis within dedicated niches. More specifically, conditions that lead to increased catecholamine levels in the host, such as stress and therapeutic treatments, have the potential to increase the risk of bacterial infections [33].

#### Uptake of ferrous iron

Acquisition systems of free ferrous iron (predominant in environments with low oxygen and/or low pH) is facilitated by the Feo (*fe*rrous ir*o*n)—ABC transporter, which has been extensively characterized in members of the *Enterobacteriaceae* family. For *Staphylococcus* spp., homologs of *feoAB* have been identified but to date, the *feoAB*(*C*) locus remains poorly characterized in Gram-positive pathogens [33] and has only recently been experimentally investigated in *S. aureus* [101] and *S. lugdunensis*^H^ [63]. For the latter study, this transport system was shown to result in the acquisition of iron under in vitro acidic culture conditions. The transport of free ferrous iron across the cytoplasmic membrane by the *s*taphylococcal iron *t*ransporter (sitABC), initially discovered in *S. epidermidis*^H^ [2, 102, 103] has been proposed. The three-gene, *sitABC*, operon is transcriptionally regulated by the diphtheria toxin repressor (DtxR)-homologue, SirR, an iron-dependent transcriptional repressor and functional homolog of Fur first discovered in a study by Hill et al. [102]. A study by Vermassen et al. [69] found an increased expression of the three *sit* genes in *S. xylosus*^H^ when grown in media with ferrous sulphate revealing their potential role in the acquisition of ferrous iron.

## Potential relevance for protective effects of NASM

Regarding the potential protective effects of NASM against major mastitis pathogens such as *S. aureus*, *Streptococcus uberis* and *E. coli* the role of siderophores in probiotic bacteria remains relatively unexplored. The ability of NASM to usurp siderophores produced by *S. aureus* could potentially reduce the colonization of *S. aureus* on the bovine teat apex or the mammary gland through siderophore-mediated iron competition [31]. For example, Deriu et al. [104] observed a probiotic *E. coli* strain found in the human gut, to outcompete and reduce *Salmonella enterica* subs. *enterica* serovar Typhimurium intestinal colonization in mice through siderophore-mediated iron competition. Interestingly, some isolates (strains) of *S. chromogenes* from Canada [11], Belgium and Norway [39] had BLAST hits for the *sirABC* genes (Additional file 1 and Table 2), indicating their potential to acquire SB from *S. aureus* as well. On the other hand, a recent study [105] highlighted the ability of siderophore-producing species, such as *S. warneri* and *M. sciuri*, isolated from human nasal cavity, to restrict iron access of *S aureus*. They achieve this by encoding unique siderophore biosynthesis clusters that do not support *S. aureus* proliferation under iron-limited conditions. *Staphylococcus lugdunensis*, a species not usually associated with bovine intramammary infection although occasionally isolated from a subclinical mastitic cow, buffalo, goat, and sheep samples in a study in Egypt [106], is known to obtain SA and SB from *S. aureus* [63]. A recent study [107] has demonstrated that siderophore-based interactions in the human nasal microbiome might partially explain the negative correlation between *S. lugdunensis* and *S. aureus*, as well as its positive correlation with other commensal NASM species. These interactions likely facilitate mutual support among commensal species while inhibiting the growth of *S. aureus*, a mechanism that could potentially be applied to the protection of the mammary gland. However, siderophore consumption and production tend to be rather strain-specific and not species-dependent. These interactions can vary depending on bacterial genotypes, particularly when factoring in other traits like bacteriocin production, which is also strain-dependent and influences bacterial dynamics.

Given the rise of antimicrobial resistance, leveraging iron acquisition systems to the detriment of pathogenic bacteria present a potential intriguing therapeutic alternative in tackling bacterial infections, also in dairy cows. This typically involves one of three approaches: (1) employing substrate-binding proteins as vaccine antigens (e.g. *isdB* and *FhuD2*), (2) inhibiting siderophore biosynthetic pathways, or (3) utilizing siderophore-uptake pathways to actively transport toxic molecules, a strategy known as the “Trojan horse” approach, as discussed in the review by Sheldon and Heinrichs [33].

A study by Misra et al. [108], supports the concept that targeting iron acquisition systems, specifically the Isd system, can be a viable vaccine strategy. The conservation and expression of IsdA in bovine-associated *S. aureus*, *S. haemolyticus, S. chromogenes*, along with its role in immune evasion and iron uptake, underscore its potential as a surface-exposed antigen for mastitis vaccine development. However, it is important to note that there is considerable variability in the detection of the *isdA* gene among these NASM species (Additional file 1 and Table 2). In a review on the critical appraisal of mastitis vaccines for dairy cows, protection conferred by Isd antigens in cattle has not yet been demonstrated [109]. Developing suitable experimental models will be essential for confirming the role of surface proteins in vivo and for enhancing current systems used to evaluate potential therapeutic and vaccine candidates [110].

Regarding the second therapeutic alternative approach, targeting siderophore biosynthetic pathways, a review by Ezzeddine and Ghssein [111], underscores the therapeutic potential of disrupting siderophore synthesis and associated transport systems. Such interventions could significantly reduce the virulence of bacteria that rely on metallophores for survival in the host environment. The authors emphasize that advancing research in this area holds strong promise for developing novel strategies to counteract antimicrobial resistance.

## Concluding remarks

Staphylococci have evolved a plethora of mechanisms to combat the various ways the host sequesters iron. When compared to *S. aureus*, there is paucity of information regarding iron acquisition in NASM over all hosts at the geno- and phenotypic levels. This area should be further investigated, including the potential protective effects of certain strains of NASM species against major (bovine mastitis) pathogens. Additionally, specifically for bovine mastitis, very little is known about bacterial iron scavenging during mammary gland infections. It remains to be determined which metabolic pathways predominate in milk and which, if any, siderophores are produced. Extensively identifying the iron uptake genes as done for *S. aureus* is essential in understanding NASM pathogenesis whether it would be for nosocomial blood stream infections in humans and/or intramammary infections in dairy cows. The development of new tools and performing existing phenotypical iron assays on bovine-associated NASM will allow to link these data with the presence/absence of genetic mediators through WGS. Furthermore, increased availability of WGS will also allow de novo prediction and identification of (distinct) iron-related proteins using AI-driven protein prediction tools such as AlphaFold2 and AlphaFill [120]. Together this will contribute to a better understanding of the relevance of certain NASM species and strains in bovine mastitis, the differences in virulence factor production and pathogenicity, as well as their probiotic potential.

## Supplementary Information

**Additional file 1. Genetic loci involved in heme- and siderophore-related iron acquisition in bovine-associated non-*aureus* staphylococci and mammaliicocci. **Values within cells represent the percentage of isolates containing the given gene. Species abbreviations: S. AGN, *S. agnetis*; S. ARL*, S. arlettae*; S. AUR, *S. auricularis*; S. BOR, *S. borealis*; S. CAPI, *S. capitis*; S. CAPR, *S. caprae*; S. CHR, *S. chromogenes*; S. COH, *S. cohnii*; S. DEV, *S. devriesei*; S. EPI, *S. epidermidis*; S. EQU, *S. equorum*; M. FLE, *M. fleurettii*; S. GAL, *S. gallinarum*; S. HAE, *S. haemolyticus*; S. HOM, *S. hominis*; S. HYI, *S. hyicus*; S. KLO, *S. kloosii*; S. NEP, *S. nepalensis*; S. PAS. S. *pasteuri*; S. SAP, *S. saprophyticus*; M. SCI, *M. sciuri*; S. SIM, *S. simulans*; S. SUC, *S. succinus*; M. VIT. *M. vitulinus*; S. WAR, *S. warneri*; and S. XYL, *S. xylosus*. ^1^Bovine-associated NASM isolates (*n* = 20) from a study by Åvall-Jääskeläinen et al. [16]. ^2^Number of isolates, ^3^Bovine-associated NASM isolates (*n* = 441) from a study by Naushad et al. [11], ^4^Bovine-associated NASM isolates (*n* = 50) from a study by Fergestad et al. [39], ^5^Bovine-associated NASM isolates (*n* = 223) from a study by Waller et al. [40], and ^6^Bovine-associated NASM isolates (*n* = 6) from a study by Reydams et al. [36], including three *S. simulans* isolates from the M-team repository used in a study by Toledo-Silva et al*.* [41].

**Additional file 2. Data set of staphylococcal iron-acquisition virulence factor sequences.**

## Data Availability

All data generated or analyzed during this study are included in this published article and its supplementary information files. Three isolates from the manuscript by Toledo-Silva et al*.* [40] have not been released, due to intellectual property (IP) protection.

## References

[1] The List of Prokaryotic names with standing in nomenclature (LSPN). https://www.bacterio.net/genus/staphylococcus. Accessed 2 Feb 2025.

[2] Sheldon JR, Heinrichs DE (2012) The iron-regulated staphylococcal lipoproteins. Front Cell Infect Microbiol 2:41 22919632 10.3389/fcimb.2012.00041 PMC3417571

[3] Vanderhaeghen W, Piepers S, Leroy F, Van Coillie E, Haesebrouck F, De Vliegher S (2015) Identification, typing, ecology and epidemiology of coagulase negative staphylococci associated with ruminants. Vet J 203:44–51 25467994 10.1016/j.tvjl.2014.11.001

[4] Becker K, Heilmann C, Peters G (2014) Coagulase-negative staphylococci. Clin Microbiol Rev 27:870–926 25278577 10.1128/CMR.00109-13 PMC4187637

[5] De Buck J, Ha V, Naushad S, Nobrega DB, Luby C, Middleton JR, De Vliegher S, Barkema HW (2021) Non-*aureus* staphylococci and bovine udder health: current understanding and knowledge gaps. Front Vet Sci 8:658031 33937379 10.3389/fvets.2021.658031 PMC8081856

[6] Gonzalez-Martin M, Corbera JA, Suarez-Bonnet A, Tejedor-Junco MT (2020) Virulence factors in coagulase-positive staphylococci of veterinary interest other than *Staphylococcus aureus*. Vet Q 40:118–131 32223696 10.1080/01652176.2020.1748253 PMC7178840

[7] dos Santos DC, Lange CC, Avellar-Costa P, dos Santos KRN, Brito MAVP, Giambiagi-deMarval M (2016) *Staphylococcus chromogenes*, a coagulase-negative *Staphylococcus* species that can clot plasma. J Clin Microbiol 54:1372–1375 26912749 10.1128/JCM.03139-15 PMC4844742

[8] Fox LK, Besser TE, Jackson SM (1996) Evaluation of a coagulase-negative variant of *Staphylococcus aureus* as a cause of intramammary infections in a herd of dairy cattle. J Am Vet Med Assoc 209:1143–1146 8800266

[9] Madhaiyan M, Wirth JS, Saravanan VS (2020) Phylogenomic analyses of the Staphylococcaceae family suggest the reclassification of five species within the genus *Staphylococcus* as heterotypic synonyms, the promotion of five subspecies to novel species, the taxonomic reassignment of five *Staphylococcus* species to *Mammaliicoccus* gen. nov., and the formal assignment of *Nosocomiicoccus* to the family Staphylococcaceae. Int J Syst Evol Microbiol 70:5926–5936 33052802 10.1099/ijsem.0.004498

[10] Gomes F, Henriques M (2016) Control of bovine mastitis: old and recent therapeutic approaches. Curr Microbiol 72:377–382 26687332 10.1007/s00284-015-0958-8

[11] Naushad S, Naqvi SA, Nobrega D, Luby C, Kastelic JP, Barkema HW, De Buck J (2019) Comprehensive virulence gene profiling of bovine non-*aureus* staphylococci based on whole-genome sequencing data. mSystems 4:e00098–18 30863792 10.1128/mSystems.00098-18 PMC6401416

[12] De Vliegher S, Fox LK, Piepers S, McDougall S, Barkema HW (2012) Invited review: mastitis in dairy heifers: nature of the disease, potential impact, prevention, and control. J Dairy Sci 95:1025–1040 22365187 10.3168/jds.2010-4074

[13] Bradley A (2002) Bovine mastitis: an evolving disease. Vet J 164:116–128 12359466 10.1053/tvjl.2002.0724

[14] Pol M, Ruegg PL (2007) Relationship between antimicrobial drug usage and antimicrobial susceptibility of Gram-positive mastitis pathogens. J Dairy Sci 90:262–273 17183094 10.3168/jds.S0022-0302(07)72627-9

[15] Stevens M, Piepers S, Supré K, Dewulf J, De Vliegher S (2016) Quantification of antimicrobial consumption in adult cattle on dairy herds in Flanders, Belgium, and associations with udder health, milk quality, and production performance. J Dairy Sci 99:2118–2130 26778315 10.3168/jds.2015-10199

[16] Åvall-Jääskeläinen S, Taponen S, Kant R, Paulin L, Blom J, Palva A, Koort J (2018) Comparative genome analysis of 24 bovine-associated *Staphylococcus* isolates with special focus on the putative virulence genes. PeerJ 6:e4560 29610707 10.7717/peerj.4560 PMC5880176

[17] Compton CW, Heuer C, Parker K, McDougall S (2007) Epidemiology of mastitis in pasture-grazed peripartum dairy heifers and its effects on productivity. J Dairy Sci 90:4157–4170 17699034 10.3168/jds.2006-880

[18] Piepers S, Opsomer G, Barkema HW, de Kruif A, De Vliegher S (2010) Heifers infected with coagulase-negative staphylococci in early lactation have fewer cases of clinical mastitis and higher milk production in their first lactation than noninfected heifers. J Dairy Sci 93:2014–2024 20412915 10.3168/jds.2009-2897

[19] Pain M, Wolden R, Jaén-Luchoro D, Salvà-Serra F, Iglesias BP, Karlsson R, Klingenberg C, Cavanagh JP (2020) *Staphylococcus borealis* sp. nov., isolated from human skin and blood. Int J Syst Evol Microbiol 70:6067–6078 33048039 10.1099/ijsem.0.004499

[20] Sipahi N, Kaya E, Çelik C, Pınar O (2023) The characterization and beta-lactam resistance of staphylococcal community recovered from raw bovine milk. Antibiotics 12:556 36978423 10.3390/antibiotics12030556 PMC10044537

[21] Condas LAZ, De Buck J, Nobrega DB, Carson DA, Naushad S, De Vliegher S, Zadoks RN, Middleton JR, Dufour S, Kastelic JP, Barkema HW (2017) Prevalence of non-*aureus* staphylococci species causing intramammary infections in Canadian dairy herds. J Dairy Sci 100:5592–5612 28527793 10.3168/jds.2016-12478

[22] De Visscher A, Piepers S, Haesebrouck F, De Vliegher S (2016) Intramammary infection with coagulase-negative staphylococci at parturition: species-specific prevalence, risk factors, and effect on udder health. J Dairy Sci 99:6457–6469 27236763 10.3168/jds.2015-10458

[23] Mahmmod YS, Klaas IC, Svennesen L, Pedersen K, Ingmer H (2018) Communications of *Staphylococcus aureus* and non-*aureus Staphylococcus* species from bovine intramammary infections and teat apex colonization. J Dairy Sci 101:7322–7333 29778469 10.3168/jds.2017-14311

[24] Piessens V, Van Coillie E, Verbist B, Supré K, Braem G, Van Nuffel A, De Vuyst L, Heyndrickx M, De Vliegher S (2011) Distribution of coagulase-negative *Staphylococcus* species from milk and environment of dairy cows differs between herds. J Dairy Sci 94:2933–2944 21605763 10.3168/jds.2010-3956

[25] National Mastitis Council (NMC) (2011) Recommended mastitis control program. https://www.nmconline.org/docs/NMCchecklistInt.pdf. Accessed Feb 2024

[26] Beuckelaere L, De Visscher A, Souza FN, Meyer E, Haesebrouck F, Piepers S, De Vliegher S (2021) Colonization and local host response following intramammary *Staphylococcus chromogenes* challenge in dry cows. Vet Res 52:137 34711282 10.1186/s13567-021-01007-8 PMC8554945

[27] Carlson SK, Erickson DL, Wilson E (2020) *Staphylococcus aureus* metal acquisition in the mastitic mammary gland. Microb Pathog 144:104179 32244043 10.1016/j.micpath.2020.104179

[28] Conroy BS, Grigg JC, Kolesnikov M, Morales LD, Murphy MEP (2019) *Staphylococcus aureus* heme and siderophore-iron acquisition pathways. Biometals 32:409–424 30911924 10.1007/s10534-019-00188-2

[29] Ghssein G, Brutesco C, Ouerdane L, Fojcik C, Izaute A, Wang S, Hajjar C, Lobinski R, Lemaire D, Richaud P, Voulhoux R, Espaillat A, Cava F, Pignol D, Borezée-Durant E, Arnoux P (2016) Biosynthesis of a broad-spectrum nicotianamine-like metallophore in *Staphylococcus aureus*. Science 352:1105–1109 27230378 10.1126/science.aaf1018

[30] Ghssein G, Ezzeddine Z (2022) The key element role of metallophores in the pathogenicity and virulence of *Staphylococcus aureus*: a review. Biology 11:1525 36290427 10.3390/biology11101525 PMC9598555

[31] Kramer J, Özkaya Ö, Kümmerli R (2020) Bacterial siderophores in community and host interactions. Nat Rev Microbiol 18:152–163 31748738 10.1038/s41579-019-0284-4 PMC7116523

[32] Marchetti M, De Bei O, Bettati S, Campanini B, Kovachka S, Gianquinto E, Spyrakis F, Ronda L (2020) Iron metabolism at the interface between host and pathogen: from nutritional immunity to antibacterial development. Int J Mol Sci 21:2145 32245010 10.3390/ijms21062145 PMC7139808

[33] Sheldon JR, Heinrichs DE (2015) Recent developments in understanding the iron acquisition strategies of Gram-positive pathogens. FEMS Microbiol Rev 39:592–630 25862688 10.1093/femsre/fuv009

[34] Sheldon JR, Laakso HA, Heinrichs DE (2016) Iron acquisition strategies of bacterial pathogens. Microbiol Spectr 4:43–85 10.1128/microbiolspec.VMBF-0010-2015 27227297

[35] van Dijk MC, de Kruijff RM, Hagedoorn PL (2022) The role of iron in *Staphylococcus aureus* infection and human disease: a metal tug of war at the host-microbe interface. Front Cell Dev Biol 10:857237 35399529 10.3389/fcell.2022.857237 PMC8986978

[36] Reydams H, Toledo-Silva B, Mertens K, Piepers S, Vereecke N, Souza FN, Haesebrouck F, De Vliegher S (2024) Phenotypic and genotypic assessment of iron acquisition in diverse bovine-associated non-*aureus staphylococcal* strains. Vet Res 55:6 38217046 10.1186/s13567-023-01260-z PMC10785429

[37] Reydams H, Wuytack A, Piepers S, Mertens K, Boyen F, de Souza FN, Haesebrouck F, De Vliegher S (2022) Genetic diversity and iron metabolism of *Staphylococcus hominis* isolates originating from bovine quarter milk, rectal feces, and teat apices. J Dairy Sci 105:9995–10006 36270870 10.3168/jds.2022-22216

[38] Wuytack A, De Visscher A, Piepers S, Boyen F, Haesebrouck F, De Vliegher S (2019) Non-*aureus staphylococci* in fecal samples of dairy cows: first report and phenotypic and genotypic characterization. J Dairy Sci 102:9345–9359 31421888 10.3168/jds.2019-16662

[39] Fergestad ME, Touzain F, De Vliegher S, De Visscher A, Thiry D, Ngassam Tchamba C, Mainil JG, L’Abee-Lund T, Blanchard Y, Wasteson Y (2021) Whole genome sequencing of staphylococci isolated from bovine milk samples. Front Microbiol 12:715851 34987483 10.3389/fmicb.2021.715851 PMC8721127

[40] Waller KP, Myrenas M, Borjesson S, Kim H, Widerstrom M, Monsen T, Siguretharson Sandholt AK, Ostlund E, Cha W (2023) Genotypic characterization of *Staphylococcus chromogenes* and *Staphylococcus simulans* from Swedish cases of bovine subclinical mastitis. J Dairy Sci 106:7991–8004 37641317 10.3168/jds.2023-23523

[41] Toledo-Silva B, de Souza FN, Mertens K, Piepers S, Haesebrouck F, De Vliegher S (2021) Bovine-associated non-*aureus staphylococci* suppress *Staphylococcus aureus* biofilm dispersal *in vitro* yet not through *agr* regulation. Vet Res 52:114 34479647 10.1186/s13567-021-00985-z PMC8414718

[42] Schaible UE, Kaufmann SH (2004) Iron and microbial infection. Nat Rev Microbiol 2:946–951 15550940 10.1038/nrmicro1046

[43] Imlay JA, Linn S (1988) DNA damage and oxygen radical toxicity. Science 240:1302–1309 3287616 10.1126/science.3287616

[44] Krewulak KD, Vogel HJ (2008) Structural biology of bacterial iron uptake. Biochim Biophys Acta 1778:1781–1804 17916327 10.1016/j.bbamem.2007.07.026

[45] MacKenzie EL, Iwasaki K, Tsuji Y (2008) Intracellular iron transport and storage: from molecular mechanisms to health implications. Antioxid Redox Signal 10:997–1030 18327971 10.1089/ars.2007.1893 PMC2932529

[46] Wardman P, Candeias LP (1996) Fenton chemistry: an introduction. Radiat Res 145:523–531 8619017

[47] Maresso AW, Schneewind O (2006) Iron acquisition and transport in *Staphylococcus aureus*. Biometals 19:193–203 16718604 10.1007/s10534-005-4863-7

[48] Wooldridge KG, Williams PH (1993) Iron uptake mechanisms of pathogenic bacteria. FEMS Microbiol Rev 12:325–348 8268005 10.1111/j.1574-6976.1993.tb00026.x

[49] Macara IG, Hoy TG, Harrison PM (1973) The formation of ferritin from apoferritin. Inhibition and metal ion-binding studies. Biochem J 135:785–788 4798313 10.1042/bj1350785 PMC1165895

[50] Srole DN, Ganz T (2021) Erythroferrone structure, function, and physiology: iron homeostasis and beyond. J Cell Physiol 236:4888–4901 33372284 10.1002/jcp.30247 PMC8026552

[51] Crichton RR, Charloteaux-Wauters M (1987) Iron transport and storage. Eur J Biochem 164:485–506 3032619 10.1111/j.1432-1033.1987.tb11155.x

[52] Kristiansen M, Graversen JH, Jacobsen C, Sonne O, Hoffman HJ, Law SK, Moestrup SK (2001) Identification of the haemoglobin scavenger receptor. Nature 409:198–201 11196644 10.1038/35051594

[53] Lim SK, Ferraro B, Moore K, Halliwell B (2001) Role of haptoglobin in free hemoglobin metabolism. Redox Rep 6:219–227 11642712 10.1179/135100001101536364

[54] Soares MP, Hamza I (2016) Macrophages and iron metabolism. Immunity 44:492–503 26982356 10.1016/j.immuni.2016.02.016 PMC4794998

[55] Nunez G, Sakamoto K, Soares MP (2018) Innate nutritional immunity. J Immunol 201:11–18 29914937 10.4049/jimmunol.1800325 PMC6028930

[56] Van Vlierberghe H, Langlois M, Delanghe J (2004) Haptoglobin polymorphisms and iron homeostasis in health and in disease. Clin Chim Acta 345:35–42 15193975 10.1016/j.cccn.2004.03.016

[57] Baker EN, Baker HM (2005) Molecular structure, binding properties and dynamics of lactoferrin. Cell Mol Life Sci 62:2531–2539 16261257 10.1007/s00018-005-5368-9 PMC11139133

[58] Rainard P (1986) Bacteriostasis of *Escherichia coli* by bovine lactoferrin, transferrin and immunoglobulins (IgG1, IgG2, IgM) acting alone or in combination. Vet Microbiol 11:103–115 3518222 10.1016/0378-1135(86)90011-8

[59] Cramer E, Pryzwansky KB, Villeval JL, Testa U, Breton-Gorius J (1985) Ultrastructural localization of lactoferrin and myeloperoxidase in human neutrophils by immunogold. Blood 65:423–458 2981589

[60] Sandrini SM, Shergill R, Woodward J, Muralikuttan R, Haigh RD, Lyte M, Freestone PP (2010) Elucidation of the mechanism by which catecholamine stress hormones liberate iron from the innate immune defense proteins transferrin and lactoferrin. J Bacteriol 192:587–594 19820086 10.1128/JB.01028-09 PMC2805316

[61] Ghssein G, Matar SF (2018) Chelating mechanisms of transition metals by bacterial metallophores “pseudopaline and staphylopine”: a quantum chemical assessment. Computation 6:56

[62] Davidson AL, Dassa E, Orelle C, Chen J (2008) Structure, function, and evolution of bacterial ATP-binding cassette systems. Microbiol Mol Biol Rev 72:317–364 18535149 10.1128/MMBR.00031-07 PMC2415747

[63] Flannagan RS, Brozyna JR, Kumar B, Adolf LA, Power JJ, Heilbronner S, Heinrichs DE (2022) In vivo growth of *Staphylococcus lugdunensis* is facilitated by the concerted function of heme and non-heme iron acquisition mechanisms. J Biol Chem 298:101823 35283192 10.1016/j.jbc.2022.101823 PMC9052147

[64] Toledo-Silva B, de Souza FN, Piepers S, Mertens K, Haesebrouck F, De Vliegher S (2021) Metabolites of bovine-associated non-*aureus staphylococci* influence expression of *Staphylococcus aureus* agr-related genes in vitro. Vet Res 52:62 33926572 10.1186/s13567-021-00933-x PMC8082617

[65] Escolar L, Perez-Martin J, de Lorenzo V (1999) Opening the iron box: transcriptional metalloregulation by the Fur protein. J Bacteriol 181:6223–6229 10515908 10.1128/jb.181.20.6223-6229.1999 PMC103753

[66] Hantke K (2001) Iron and metal regulation in bacteria. Curr Opin Microbiol 4:172–177 11282473 10.1016/s1369-5274(00)00184-3

[67] Beasley FC, Heinrichs DE (2010) Siderophore-mediated iron acquisition in the staphylococci. J Inorg Biochem 104:282–288 19850350 10.1016/j.jinorgbio.2009.09.011

[68] Braun V (2001) Iron uptake mechanisms and their regulation in pathogenic bacteria. Int J Med Microbiol 291:67–79 11437341 10.1078/1438-4221-00103

[69] Vermassen A, Talon R, Leroy S (2016) Ferritin, an iron source in meat for *Staphylococcus xylosus*? Int J Food Microbiol 225:20–26 26971013 10.1016/j.ijfoodmicro.2016.03.005

[70] Skaar EP, Humayun M, Bae T, DeBord KL, Schneewind O (2004) Iron-source preference of *Staphylococcus aureus* infections. Science 305:1626–1629 15361626 10.1126/science.1099930

[71] Grigg JC, Ukpabi G, Gaudin CF, Murphy ME (2010) Structural biology of heme binding in the *Staphylococcus aureus* Isd system. J Inorg Biochem 104:341–348 19853304 10.1016/j.jinorgbio.2009.09.012

[72] Hammer ND, Skaar EP (2011) Molecular mechanisms of *Staphylococcus aureus* iron acquisition. Annu Rev Microbiol 65:129–147 21639791 10.1146/annurev-micro-090110-102851 PMC3807827

[73] Madigan CA, Martinot AJ, Wei JR, Madduri A, Cheng TY, Young DC, Layre E, Murry JP, Rubin EJ, Moody DB (2015) Lipidomic analysis links mycobactin synthase K to iron uptake and virulence in *Mycobacterium tuberculosis*. PLoS Pathog 11:e1004792 25815898 10.1371/journal.ppat.1004792 PMC4376628

[74] Farrand AJ, Haley KP, Lareau NM, Heilbronner S, McLean JA, Foster T, Skaar EP (2015) An iron-regulated autolysin remodels the cell wall to facilitate heme acquisition in *Staphylococcus lugdunensis*. Infect Immun 83:3578–3589 26123800 10.1128/IAI.00397-15 PMC4534641

[75] Haley KP, Janson EM, Heilbronner S, Foster TJ, Skaar EP (2011) *Staphylococcus lugdunensis* IsdG liberates iron from host heme. J Bacteriol 193:4749–4757 21764939 10.1128/JB.00436-11 PMC3165669

[76] Zapotoczna M, Heilbronner S, Speziale P, Foster TJ (2012) Iron-regulated surface determinant (Isd) proteins of *Staphylococcus lugdunensis*. J Bacteriol 194:6453–6467 23002220 10.1128/JB.01195-12 PMC3497513

[77] Sun ZW, Zhou DY, Zhang XY, Li QL, Lin HL, Lu W, Liu HM, Lu JW, Lin X, Li KW, Xu T, Bao QY, Zhang HL (2020) Determining the genetic characteristics of resistance and virulence of the “*Epidermidis* cluster group” through pan-genome analysis. Front Cell Infect Microbiol 10:274 32596166 10.3389/fcimb.2020.00274 PMC7303328

[78] Beasley FC, Vines ED, Grigg JC, Zheng Q, Liu S, Lajoie GA, Murphy ME, Heinrichs DE (2009) Characterization of staphyloferrin A biosynthetic and transport mutants in *Staphylococcus aureus*. Mol Microbiol 72:947–963 19400778 10.1111/j.1365-2958.2009.06698.x

[79] Cotton JL, Tao J, Balibar CJ (2009) Identification and characterization of the *Staphylococcus aureus* gene cluster coding for staphyloferrin A. Biochemistry 48:1025–1035 19138128 10.1021/bi801844c

[80] Cheung J, Beasley FC, Liu S, Lajoie GA, Heinrichs DE (2009) Molecular characterization of staphyloferrin B biosynthesis in *Staphylococcus aureus*. Mol Microbiol 74:594–608 19775248 10.1111/j.1365-2958.2009.06880.x

[81] Dale SE, Doherty-Kirby A, Lajoie G, Heinrichs DE (2004) Role of siderophore biosynthesis in virulence of *Staphylococcus aureus*: identification and characterization of genes involved in production of a siderophore. Infect Immun 72:29–37 14688077 10.1128/IAI.72.1.29-37.2004 PMC343950

[82] Sheldon JR, Marolda CL, Heinrichs DE (2014) TCA cycle activity in *Staphylococcus aureus* is essential for iron-regulated synthesis of staphyloferrin A, but not staphyloferrin B: the benefit of a second citrate synthase. Mol Microbiol 92:824–839 24666349 10.1111/mmi.12593

[83] Ben Zakour NL, Beatson SA, van den Broek AH, Thoday KL, Fitzgerald JR (2012) Comparative genomics of the *Staphylococcus intermedius* group of animal pathogens. Front Cell Infect Microbiol 2:44 22919635 10.3389/fcimb.2012.00044 PMC3417630

[84] Kobylarz MJ, Grigg JC, Sheldon JR, Heinrichs DE, Murphy ME (2014) SbnG, a citrate synthase in *Staphylococcus aureus*: a new fold on an old enzyme. J Biol Chem 289:33797–33807 25336653 10.1074/jbc.M114.603175 PMC4256314

[85] Brozyna JR, Sheldon JR, Heinrichs DE (2014) Growth promotion of the opportunistic human pathogen, *Staphylococcus lugdunensis*, by heme, hemoglobin, and coculture with *Staphylococcus aureus*. Microbiol Open 3:182–195 10.1002/mbo3.162 PMC3996567 24515974

[86] Grigg JC, Cheung J, Heinrichs DE, Murphy ME (2010) Specificity of staphyloferrin B recognition by the SirA receptor from *Staphylococcus aureus*. J Biol Chem 285:34579–34588 20810662 10.1074/jbc.M110.172924 PMC2966073

[87] Grigg JC, Cooper JD, Cheung J, Heinrichs DE, Murphy ME (2010) The *Staphylococcus aureus* siderophore receptor HtsA undergoes localized conformational changes to enclose staphyloferrin A in an arginine-rich binding pocket. J Biol Chem 285:11162–11171 20147287 10.1074/jbc.M109.097865 PMC2856993

[88] Skaar EP, Gaspar AH, Schneewind O (2004) IsdG and IsdI, heme-degrading enzymes in the cytoplasm of *Staphylococcus aureus*. J Biol Chem 279:436–443 14570922 10.1074/jbc.M307952200

[89] Oliveira F, Lima T, Correia A, Silva AM, Soares C, Morais S, Weisselberg S, Vilanova M, Rohde H, Cerca N (2021) Siderophore-mediated iron acquisition plays a critical role in biofilm formation and survival of *Staphylococcus epidermidis* within the host. Front Med 8:799227 10.3389/fmed.2021.799227 PMC8738164 35004774

[90] Lindsay JA, Riley TV, Mee BJ (1995) *Staphylococcus aureus* but not *Staphylococcus epidermidis* can acquire iron from transferrin. Microbiology (Reading) 141:197–203 7894712 10.1099/00221287-141-1-197

[91] Modun B, Evans RW, Joannou CL, Williams P (1998) Receptor-mediated recognition and uptake of iron from human transferrin by *Staphylococcus aureus* and *Staphylococcus epidermidis*. Infect Immun 66:3591–3596 9673237 10.1128/iai.66.8.3591-3596.1998 PMC108390

[92] Modun BJ, Cockayne A, Finch R, Williams P (1998) The *Staphylococcus aureus* and *Staphylococcus epidermidis* transferrin-binding proteins are expressed in vivo during infection. Microbiology 144:1005–1012 9579074 10.1099/00221287-144-4-1005

[93] Sebulsky MT, Hohnstein D, Hunter MD, Heinrichs DE (2000) Identification and characterization of a membrane permease involved in iron-hydroxamate transport in *Staphylococcus aureus*. J Bacteriol 182:4394–4400 10913070 10.1128/jb.182.16.4394-4400.2000 PMC94608

[94] Beasley FC, Marolda CL, Cheung J, Buac S, Heinrichs DE (2011) *Staphylococcus aureus* transporters Hts, Sir, and Sst capture iron liberated from human transferrin by staphyloferrin A, staphyloferrin B, and catecholamine stress hormones, respectively, and contribute to virulence. Infect Immun 79:2345–2355 21402762 10.1128/IAI.00117-11 PMC3125851

[95] Sebulsky MT, Heinrichs DE (2001) Identification and characterization of *fhuD1* and *fhuD2*, two genes involved in iron-hydroxamate uptake in *Staphylococcus aureus*. J Bacteriol 183:4994–5000 11489851 10.1128/JB.183.17.4994-5000.2001 PMC95374

[96] Sebulsky MT, Shilton BH, Speziali CD, Heinrichs DE (2003) The role of FhuD2 in iron(III)-hydroxamate transport in *Staphylococcus**aureus*. Demonstration that FhuD2 binds iron(III)-hydroxamates but with minimal conformational change and implication of mutations on transport. J Biol Chem 278:49890–49900 14514690 10.1074/jbc.M305073200

[97] Freestone PPE, Haigh RD, Williams PH, Lyte M (1999) Stimulation of bacterial growth by heat-stable, norepinephrine-induced autoinducers. FEMS Microbiol Lett 172:53–60 10079527 10.1111/j.1574-6968.1999.tb13449.x

[98] Neal CP, Freestone PPE, Maggs AF, Haigh RD, Williams PH, Lyte M (2001) Catecholamine inotropes as growth factors for *Staphylococcus epidermidis* and other coagulase-negative staphylococci. FEMS Microbiol Lett 194:163–169 11164302 10.1111/j.1574-6968.2001.tb09463.x

[99] Lyte M, Freestone PP, Neal CP, Olson BA, Haigh RD, Bayston R, Williams PH (2003) Stimulation of *Staphylococcus epidermidis* growth and biofilm formation by catecholamine inotropes. Lancet 361:130–135 12531580 10.1016/S0140-6736(03)12231-3

[100] Freestone PPE, Sandrini SM, Haigh RD, Lyte M (2008) Microbial endocrinology: how stress influences susceptibility to infection. Trends Microbiol 16:55–64 18191570 10.1016/j.tim.2007.11.005

[101] Shin M, Jin Y, Park J, Mun D, Kim SR, Payne SM, Kim KH, Kim Y (2021) Characterization of an antibacterial agent targeting ferrous iron transport protein FeoB against *Staphylococcus aureus* and gram-positive bacteria. ACS Chem Biol 16:136–149 33378170 10.1021/acschembio.0c00842

[102] Hill PJ, Cockayne A, Landers P, Morrissey JA, Sims CM, Williams P (1998) Sirr, a novel iron-dependent repressor in *Staphylococcus epidermidis*. Infect Immun 66:4123–4129 9712757 10.1128/iai.66.9.4123-4129.1998 PMC108495

[103] Massonet C, Pintens V, Merckx R, Anné J, Lammertyn E, Van Eldere J (2006) Effect of iron on the expression of sirR and sitABC in biofilm-associated *Staphylococcus epidermidis*. BMC Microbiol 6:103 17177984 10.1186/1471-2180-6-103 PMC1764749

[104] Deriu E, Liu JZ, Pezeshki M, Edwards RA, Ochoa RJ, Contreras H, Libby SJ, Fang FC, Raffatellu M (2013) Probiotic bacteria reduce *Salmonella Typhimurium* intestinal colonization by competing for iron. Cell Host Microbe 14:26–37 23870311 10.1016/j.chom.2013.06.007 PMC3752295

[105] Zhao Y, Bitzer A, Power JJ, Belikova D, Torres Salazar BO, Adolf LA, Gerlach D, Krismer B, Heilbronner S (2024) Nasal commensals reduce *Staphylococcus aureus* proliferation by restricting siderophore availability. ISME J 18:wrae123 38987933 10.1093/ismejo/wrae123 PMC11296517

[106] El-Jakee JK, Aref NE, Gomaa A, El-Hariri MD, Galal HM, Omar SA, Samir A (2013) Emerging of coagulase-negative staphylococci as a cause of mastitis in dairy animals: an environmental hazard. Int J Vet Sci Med 1:74–78

[107] Rosenstein R, Salazar BOT, Sauer C, Heilbronner S, Krismer B, Peschel A (2024) Siderophore piracy enables the nasal commensal *Staphylococcus lugdunensis* to antagonize the pathogen *Staphylococcus aureus*. bioRxiv 10.1101/2024.02.29.582731

[108] Misra N, Wines TF, Knopp CL, McGuire MA, Tinker JK (2017) Expression, immunogenicity and variation of iron-regulated surface protein A from bovine isolates of *Staphylococcus aureus*. FEMS Microbiol Lett 364:fnx082 28430959 10.1093/femsle/fnx082 PMC5430615

[109] Rainard P, Gilbert FB, Germon P, Foucras G (2021) Invited review: a critical appraisal of mastitis vaccines for dairy cows. J Dairy Sci 104:10427–10448 34218921 10.3168/jds.2021-20434

[110] Pickering AC, Fitzgerald JR (2020) The role of Gram-positive surface proteins in bacterial niche- and host-specialization. Front Microbiol 11:2020 33193271 10.3389/fmicb.2020.594737 PMC7658395

[111] Ezzeddine Z, Ghssein G (2023) Towards new antibiotic classes targeting bacterial metallophores. Microb Pathog 182:106221 37391099 10.1016/j.micpath.2023.106221

[112] Adolf LA (2022) Structural organization and energization of iron acquisition systems in staphylococcal membranes. PhD Thesis, University of Tübingen

[113] Konetschny-Rapp S, Jung G, Meiwes J, Zähner H (1990) Staphyloferrin a: a structurally new siderophore from staphylococci. Eur J Biochem 191:65–74 2379505 10.1111/j.1432-1033.1990.tb19094.x

[114] Meiwes J, Fiedler HP, Haag H, Zahner H, Konetschny-Rapp S, Jung G (1990) Isolation and characterization of staphyloferrin A, a compound with siderophore activity from *Staphylococcus hyicus* DSM 20459. FEMS Microbiol Lett 67:201–205 10.1111/j.1574-6968.1990.tb13863.x 2139423

[115] Wilcox MH, Williams P, Smith DG, Modun B, Finch RG, Denyer SP (1991) Variation in the expression of cell envelope proteins of coagulase-negative staphylococci cultured under iron-restricted conditions in human peritoneal dialysate. J Gen Microbiol 137:2561–2570 1783903 10.1099/00221287-137-11-2561

[116] Modun B, Williams P, Pike WJ, Cockayne A, Arbuthnott JP, Finch R, Denyer SP (1992) Cell envelope proteins of *Staphylococcus epidermidis* grown *in vivo* in a peritoneal chamber implant. Infect Immun 60:2551–2553 1587623 10.1128/iai.60.6.2551-2553.1992 PMC257197

[117] Drechsel H, Freund S, Nicholson G, Haag H, Jung O, Zähner H, Jung G (1993) Purification and chemical characterization of staphyloferrin B, a hydrophilic siderophore from staphylococci. Biometals 6:185–192 8400765 10.1007/BF00205858

[118] Haag H, Fiedler HP, Meiwes J, Drechsel H, Jung G, Zähner H (1994) Isolation and biological characterization of staphyloferrin B, a compound with siderophore activity from staphylococci. FEMS Microbiol Lett 115:125–130 8138126 10.1111/j.1574-6968.1994.tb06626.x

[119] Lindsay JA, Riley TV (1994) Staphylococcal iron requirements, siderophore production, and iron-regulated protein expression. Infect Immun 62:2309–2314 8188353 10.1128/iai.62.6.2309-2314.1994 PMC186513

[120] Heidrich C, Hantke K, Bierbaum G, Sahl HG (1996) Identification and analysis of a gene encoding a Fur-like protein of *Staphylococcus epidermidis*. FEMS Microbiol Lett 140:253–259 8764488 10.1111/j.1574-6968.1996.tb08345.x

[121] Cockayne A, Hill PJ, Powell NB, Bishop K, Sims C, Williams P (1998) Molecular cloning of a 32-kilodalton lipoprotein component of a novel iron-regulated *Staphylococcus epidermidis* ABC transporter. Infect Immun 66:3767–3774 9673260 10.1128/iai.66.8.3767-3774.1998 PMC108413

[122] Morrissey JA, Cockayne A, Hill PJ, Williams P (2000) Molecular cloning and analysis of a putative siderophore ABC transporter from *Staphylococcus aureus*. Infect Immun 68:6281–6288 11035736 10.1128/iai.68.11.6281-6288.2000 PMC97710

[123] Heilbronner S, Holden MT, van Tonder A, Geoghegan JA, Foster TJ, Parkhill J, Bentley SD (2011) Genome sequence of *Staphylococcus lugdunensis* N920143 allows identification of putative colonization and virulence factors. FEMS Microbiol Lett 322:60–67 21682763 10.1111/j.1574-6968.2011.02339.x PMC3615170

[124] Souza BSV, Silva KCS, Parente AFA, Borges CL, Paccez JD, Pereira M, Soares CMA, Giambiagi-deMarval M, Silva-Bailao MG, Parente-Rocha JA (2019) The influence of pH on *Staphylococcus saprophyticus* iron metabolism and the production of siderophores. Microb Infect 21:456–463 10.1016/j.micinf.2019.04.008 31075417

[125] Verstraete MM, Morales LD, Kobylarz M, Loutet SA, Laakso HA, Pinter TB, Stillman MJ, Heinrichs DE, Murphy MEP (2019) The heme-sensitive regulator SbnI has a bifunctional role in staphyloferrin B production by *Staphylococcus aureus*. J Biol Chem 294:11622–11636 31197035 10.1074/jbc.RA119.007757 PMC6663872

