# Supplementary material for: Pheno- and genotypic iron acquisition of non-aureus staphylococci: a scoping review of literature with a focus on bovine mastitis
Source: Vet Res. 2025 Sep 25;56:176. doi: 10.1186/s13567-025-01613-w (PMC12465704; doi:10.1186/s13567-025-01613-w)
Supplement: Supplementary file 1 — Additional file 1. Genetic loci involved in heme- and siderophore-related iron acquisition in bovine-associated non-aureus staphylococci and mammaliicocci. Values within cells represent the percentage of isolates containing the given gene. Species abbreviations: S. AGN, S. agnetis; S. ARL, S. arlettae; S. AUR, S. auricularis; S. BOR, S. borealis; S. CAPI, S. capitis; S. CAPR, S. caprae; S. CHR, S. chromogenes; S. COH, S. cohnii; S. DEV, S. devriesei; S. EPI, S. epidermidis; S. EQU, S. equorum; M. FLE, M. fleurettii; S. GAL, S. gallinarum; S. HAE, S. haemolyticus; S. HOM, S. hominis; S. HYI, S. hyicus; S. KLO, S. kloosii; S. NEP, S. nepalensis; S. PAS. S. pasteuri; S. SAP, S. saprophyticus; M. SCI, M. sciuri; S. SIM, S. simulans; S. SUC, S. succinus; M. VIT. M. vitulinus; S. WAR, S. warneri; and S. XYL, S. xylosus. 1Bovine-associated NASM isolates (n = 20) from a study by Åvall-Jääskeläinen et al. [16]. 2Number of isolates, 3Bovine-associated NASM isolates (n = 441) from a study by Naushad et al. [11], 4Bovine-associated NASM isolates (n = 50) from a study by Fergestad et al. [39], 5Bovine-associated NASM isolates (n = 223) from a study by Waller et al. [40], and 6Bovine-associated NASM isolates (n = 6) from a study by Reydams et al. [36], including three S. simulans isolates from the M-team repository used in a study by Toledo-Silva et al. [41]. [file 13567_2025_1613_MOESM1_ESM.docx]

**Additional file 1. Genetic loci involved in heme- and siderophore-related iron acquisition in bovine-associated non-*aureus* staphylococci and mammaliicocci**

| Bovine NASM | Heme (isd) | | | | | | | | |  | Endogenous Siderophores | | | | | | | | | | | | | | | | | | | |
| --- | --- | --- | --- | --- | --- | --- | --- | --- | --- | --- | --- | --- | --- | --- | --- | --- | --- | --- | --- | --- | --- | --- | --- | --- | --- | --- | --- | --- | --- | --- |
|  |  |  |  |  |  |  |  |  |  |  | Staphyloferrin A | | | | | | |  | Staphyloferrin B | | | | | | | | | | | |
|  | Binding and extraction | | | Import | | | | Degredation | |  | Synthesis and export | | | | Import and release | | |  | Synthesis and export | | | | | | | | | Import and release | | |
|  | isdA | isdB | isdH | isdC | isdD | isdE | isdF | isdG | isdI |  | sfaA | sfaB | sfaC | sfaD | htsA | htsB | htsC |  | sbnA | sbnB | sbnC | sbnD | sbnE | sbnF | sbnG | sbnH | sbnI | sirA | sirB | sirC |
| **Finland^1^** |  |  |  |  |  |  |  |  |  |  |  |  |  |  |  |  |  |  |  |  |  |  |  |  |  |  |  |  |  |  |
| S. AGN (4)^2^ | 0 | 0 | 0 | 0 | 0 | 0 | 0 | 0 | 100 |  |  |  |  |  |  |  |  |  |  |  |  |  |  |  |  |  |  |  |  |  |
| S. CHR (8) | 38 | 0 | 0 | 0 | 0 | 0 | 0 | 0 | 100 |  |  |  |  |  |  |  |  |  |  |  |  |  |  |  |  |  |  |  |  |  |
| S. SIM (8) | 25 | 0 | 0 | 88 | 0 | 88 | 75 | 100 | 88 |  |  |  |  |  |  |  |  |  |  |  |  |  |  |  |  |  |  |  |  |  |
| **Canada^3^** |  |  |  |  |  |  |  |  |  |  |  |  |  |  |  |  |  |  |  |  |  |  |  |  |  |  |  |  |  |  |
| S. AGN (13) | 0 | 0 | 0 | 0 | 0 | 0 | 0 | 0 | 100 |  | 100 | 100 | 100 | 100 | 100 | 100 | 100 |  | 100 | 100 | 100 | 100 | 100 | 100 | 100 | 100 | 100 | 100 | 100 | 100 |
| S. ARL (15) | 0 | 0 | 0 | 0 | 0 | 0 | 0 | 100 | 0 |  | 0 | 100 | 100 | 100 | 0 | 0 | 100 |  | 100 | 93 | 93 | 93 | 93 | 93 | 93 | 93 | 93 | 87 | 100 | 100 |
| S. AUR (2) | 0 | 0 | 0 | 100 | 0 | 100 | 100 | 0 | 100 |  | 100 | 100 | 50 | 100 | 100 | 100 | 100 |  | 100 | 0 | 0 | 0 | 0 | 0 | 0 | 0 | 0 | 0 | 0 | 0 |
| S. CAPI (22) | 95 | 0 | 0 | 100 | 0 | 100 | 100 | 100 | 100 |  | 100 | 100 | 95 | 100 | 100 | 100 | 100 |  | 100 | 0 | 0 | 0 | 0 | 0 | 0 | 0 | 0 | 0 | 0 | 95 |
| S. CAPR (1) | 0 | 0 | 0 | 100 | 0 | 100 | 100 | 100 | 100 |  | 100 | 100 | 100 | 100 | 100 | 100 | 100 |  | 100 | 0 | 0 | 0 | 0 | 0 | 0 | 0 | 0 | 0 | 100 | 100 |
| S. CHR (83) | 0 | 0 | 0 | 0 | 0 | 0 | 0 | 0 | 99 |  | 100 | 100 | 99 | 100 | 99 | 100 | 100 |  | 100 | 0 | 0 | 0 | 0 | 0 | 0 | 0 | 0 | 4 | 100 | 100 |
| S. COH (24) | 0 | 0 | 0 | 0 | 0 | 0 | 38 | 4 | 100 |  | 100 | 92 | 92 | 100 | 100 | 100 | 100 |  | 100 | 42 | 0 | 0 | 4 | 0 | 0 | 0 | 0 | 0 | 0 | 67 |
| S. DEV (8) | 0 | 0 | 0 | 0 | 0 | 0 | 0 | 0 | 100 |  | 88 | 100 | 100 | 100 | 100 | 100 | 100 |  | 100 | 0 | 0 | 0 | 0 | 0 | 0 | 0 | 0 | 100 | 100 | 100 |
| S. EPI (26) | 0 | 0 | 0 | 0 | 0 | 0 | 0 | 100 | 0 |  | 100 | 100 | 100 | 100 | 100 | 100 | 100 |  | 100 | 0 | 0 | 0 | 0 | 0 | 0 | 0 | 0 | 0 | 58 | 4 |
| S. EQU (17) | 0 | 0 | 0 | 0 | 0 | 0 | 0 | 0 | 100 |  | 0 | 100 | 100 | 100 | 100 | 100 | 100 |  | 100 | 100 | 94 | 100 | 94 | 94 | 100 | 100 | 100 | 100 | 100 | 100 |
| M. FLE (2) | 0 | 0 | 0 | 0 | 0 | 0 | 100 | 0 | 100 |  | 0 | 100 | 0 | 0 | 0 | 100 | 0 |  | 100 | 0 | 0 | 0 | 0 | 0 | 0 | 100 | 0 | 100 | 0 | 100 |
| S. GAL (21) | 0 | 0 | 0 | 0 | 0 | 0 | 5 | 0 | 100 |  | 100 | 100 | 100 | 95 | 100 | 100 | 100 |  | 100 | 0 | 0 | 0 | 0 | 0 | 0 | 0 | 0 | 57 | 5 | 0 |
| S. HAE (29) | 0 | 0 | 0 | 0 | 0 | 0 | 0 | 0 | 97 |  | 100 | 100 | 76 | 100 | 93 | 100 | 100 |  | 100 | 0 | 0 | 0 | 0 | 0 | 0 | 0 | 0 | 59 | 93 | 100 |
| S. HOM (11) | 0 | 0 | 0 | 0 | 0 | 0 | 45 | 0 | 100 |  | 0 | 100 | 100 | 100 | 100 | 100 | 100 |  | 100 | 0 | 0 | 0 | 0 | 0 | 0 | 0 | 0 | 0 | 0 | 0 |
| S. HYI (3) | 0 | 0 | 0 | 0 | 0 | 0 | 0 | 0 | 100 |  | 100 | 100 | 100 | 100 | 100 | 100 | 100 |  | 100 | 100 | 100 | 100 | 100 | 100 | 100 | 100 | 100 | 100 | 100 | 100 |
| S. KLO (1) | 0 | 0 | 0 | 0 | 0 | 0 | 0 | 0 | 100 |  | 100 | 100 | 100 | 100 | 100 | 100 | 100 |  | 100 | 0 | 0 | 0 | 0 | 0 | 0 | 0 | 0 | 100 | 0 | 0 |
| S. NEP (2) | 0 | 0 | 0 | 0 | 0 | 0 | 0 | 0 | 100 |  | 100 | 100 | 100 | 100 | 100 | 100 | 100 |  | 100 | 0 | 0 | 0 | 0 | 0 | 0 | 0 | 0 | 100 | 100 | 0 |
| S. PAS (6) | 33 | 0 | 100 | 100 | 0 | 100 | 100 | 100 | 83 |  | 100 | 100 | 100 | 100 | 100 | 100 | 100 |  | 100 | 0 | 0 | 0 | 0 | 0 | 0 | 0 | 0 | 0 | 0 | 100 |
| S. SAP (16) | 0 | 0 | 0 | 0 | 0 | 0 | 0 | 0 | 100 |  | 100 | 100 | 100 | 100 | 100 | 100 | 100 |  | 100 | 0 | 0 | 0 | 0 | 0 | 0 | 0 | 0 | 100 | 0 | 0 |
| M. SCI (29) | 0 | 0 | 0 | 59 | 0 | 59 | 100 | 0 | 62 |  | 0 | 0 | 0 | 0 | 97 | 100 | 100 |  | 100 | 0 | 0 | 0 | 0 | 0 | 10 | 52 | 0 | 100 | 0 | 52 |
| S. SIM (42) | 2 | 0 | 0 | 100 | 0 | 100 | 100 | 0 | 100 |  | 100 | 100 | 100 | 100 | 100 | 100 | 100 |  | 100 | 0 | 0 | 0 | 0 | 0 | 0 | 0 | 0 | 0 | 0 | 0 |
| S. SUC (15) | 0 | 0 | 0 | 0 | 0 | 0 | 0 | 100 | 0 |  | 100 | 100 | 100 | 100 | 100 | 100 | 100 |  | 100 | 0 | 0 | 0 | 0 | 0 | 0 | 0 | 0 | 0 | 93 | 0 |
| M. VIT (6) | 0 | 0 | 0 | 0 | 0 | 0 | 100 | 0 | 100 |  | 100 | 0 | 0 | 0 | 0 | 100 | 100 |  | 100 | 0 | 0 | 0 | 0 | 0 | 0 | 100 | 0 | 100 | 0 | 100 |
| S. WAR (19) | 0 | 0 | 0 | 0 | 0 | 0 | 79 | 0 | 100 |  | 100 | 100 | 100 | 100 | 100 | 100 | 100 |  | 100 | 0 | 0 | 0 | 0 | 0 | 0 | 0 | 0 | 0 | 0 | 21 |
| S. XYL (28) | 0 | 0 | 0 | 0 | 0 | 0 | 0 | 0 | 100 |  | 100 | 96 | 100 | 100 | 100 | 100 | 100 |  | 100 | 0 | 0 | 0 | 0 | 0 | 0 | 0 | 0 | 96 | 61 | 46 |
| **Belgium/Norway^4^** |  |  |  |  |  |  |  |  |  |  |  |  |  |  |  |  |  |  |  |  |  |  |  |  |  |  |  |  |  |  |
| S. ARL (1) | 0 | 0 | 0 | 0 | 0 | 0 | 100 | 0 | 100 |  | 0 | 100 | 100 | 100 | 100 | 100 | 100 |  | 100 | 100 | 100 | 100 | 100 | 100 | 100 | 100 | 100 | 100 | 100 | 100 |
| S. AUR (1) | 0 | 0 | 0 | 0 | 0 | 0 | 100 | 100 | 100 |  | 100 | 100 | 100 | 100 | 100 | 100 | 100 |  | 100 | 0 | 0 | 0 | 0 | 0 | 0 | 0 | 0 | 100 | 100 | 0 |
| S. BOR (2) | 0 | 0 | 0 | 0 | 0 | 0 | 0 | 0 | 0 |  | 100 | 100 | 100 | 100 | 100 | 100 | 100 |  | 0 | 0 | 0 | 0 | 0 | 0 | 0 | 0 | 0 | 0 | 0 | 0 |
| S. CHR (6) | 0 | 0 | 0 | 0 | 0 | 0 | 0 | 100 | 100 |  | 100 | 100 | 100 | 100 | 100 | 100 | 100 |  | 100 | 17 | 0 | 0 | 0 | 0 | 0 | 0 | 0 | 100 | 100 | 83 |
| S. COH (2) | 0 | 0 | 0 | 0 | 0 | 0 | 100 | 0 | 100 |  | 100 | 100 | 100 | 100 | 100 | 100 | 100 |  | 100 | 100 | 0 | 0 | 0 | 0 | 0 | 0 | 0 | 100 | 100 | 0 |
| S. DEV (2) | 0 | 0 | 0 | 0 | 0 | 0 | 100 | 100 | 100 |  | 100 | 100 | 100 | 100 | 100 | 100 | 100 |  | 100 | 0 | 0 | 0 | 0 | 0 | 0 | 0 | 0 | 100 | 100 | 100 |
| S. EPI (7) | 0 | 0 | 0 | 0 | 0 | 0 | 100 | 100 | 86 |  | 100 | 100 | 100 | 100 | 100 | 100 | 100 |  | 100 | 0 | 0 | 0 | 0 | 0 | 0 | 0 | 0 | 0 | 100 | 0 |
| S. EQU (2) | 0 | 0 | 0 | 0 | 0 | 0 | 100 | 100 | 100 |  | 0 | 100 | 100 | 100 | 100 | 100 | 100 |  | 100 | 100 | 100 | 100 | 100 | 100 | 100 | 100 | 50 | 100 | 100 | 100 |
| S. HAE (9) | 0 | 0 | 0 | 0 | 0 | 0 | 89 | 0 | 0 |  | 100 | 100 | 100 | 100 | 100 | 100 | 100 |  | 100 | 0 | 0 | 0 | 0 | 0 | 0 | 0 | 0 | 100 | 100 | 100 |
| S. HOM (4) | 0 | 0 | 0 | 0 | 0 | 0 | 0 | 100 | 100 |  | 0 | 100 | 100 | 75 | 25 | 100 | 100 |  | 100 | 0 | 0 | 0 | 0 | 0 | 0 | 0 | 0 | 0 | 0 | 0 |
| S. HYI (1) | 0 | 0 | 0 | 0 | 0 | 0 | 100 | 100 | 100 |  | 100 | 100 | 100 | 100 | 100 | 100 | 100 |  | 100 | 100 | 100 | 100 | 100 | 100 | 100 | 100 | 100 | 100 | 100 | 100 |
| S. SAP (1) | 0 | 0 | 0 | 0 | 0 | 0 | 0 | 100 | 100 |  | 100 | 100 | 100 | 100 | 100 | 100 | 100 |  | 100 | 0 | 0 | 0 | 0 | 0 | 0 | 0 | 0 | 0 | 100 | 100 |
| M. SCI (5) | 0 | 0 | 0 | 0 | 0 | 0 | 100 | 0 | 0 |  | 0 | 0 | 0 | 0 | 40 | 40 | 100 |  | 100 | 0 | 0 | 0 | 0 | 0 | 0 | 0 | 0 | 40 | 40 | 0 |
| S. SIM (2) | 0 | 0 | 0 | 0 | 0 | 0 | 100 | 100 | 100 |  | 100 | 100 | 100 | 100 | 100 | 100 | 100 |  | 100 | 0 | 0 | 0 | 0 | 0 | 0 | 0 | 0 | 0 | 100 | 100 |
| M. VIT (1) | 0 | 0 | 0 | 0 | 0 | 0 | 100 | 0 | 100 |  | 0 | 0 | 0 | 0 | 100 | 100 | 100 |  | 100 | 0 | 0 | 0 | 0 | 0 | 0 | 100 | 0 | 100 | 0 | 100 |
| S. WAR (2) | 0 | 0 | 0 | 0 | 0 | 0 | 100 | 100 | 100 |  | 100 | 100 | 100 | 100 | 100 | 100 | 100 |  | 100 | 0 | 0 | 0 | 0 | 0 | 0 | 0 | 0 | 100 | 100 | 100 |
| S. XYL (2) | 0 | 0 | 0 | 0 | 0 | 0 | 100 | 100 | 100 |  | 100 | 100 | 100 | 100 | 100 | 100 | 100 |  | 100 | 0 | 0 | 0 | 0 | 0 | 0 | 0 | 0 | 100 | 100 | 0 |
| **Sweden^5^** |  |  |  |  |  |  |  |  |  |  |  |  |  |  |  |  |  |  |  |  |  |  |  |  |  |  |  |  |  |  |
| S. CHR (105) | 0 | 0 | 0 | 0 | 0 | 0 | 0 | 0 | 0 |  | 100 | 100 | 100 | 100 | 100 | 100 | 100 |  | 100 | 0 | 0 | 0 | 0 | 0 | 0 | 0 | 0 | 5 | 100 | 98 |
| S. SIM (118) | 2 | 5 | 100 | 0 | 100 | 56 | 0 | 0 | 100 |  | 100 | 100 | 100 | 100 | 82 | 100 | 100 |  | 100 | 0 | 0 | 0 | 0 | 0 | 0 | 0 | 0 | 0 | 0 | 0 |
| **Belgium^6^** |  |  |  |  |  |  |  |  |  |  |  |  |  |  |  |  |  |  |  |  |  |  |  |  |  |  |  |  |  |  |
| S. CHR (4) | 0 | 0 | 0 | 0 | 0 | 0 | 0 | 0 | 100 |  | 100 | 100 | 100 | 100 | 100 | 100 | 100 |  | 0 | 0 | 0 | 0 | 0 | 0 | 0 | 0 | 0 | 0 | 100 | 100 |
| S. EQU (2) | 0 | 0 | 0 | 0 | 0 | 0 | 0 | 0 | 0 |  | 0 | 100 | 100 | 100 | 100 | 100 | 100 |  | 100 | 100 | 100 | 100 | 100 | 100 | 100 | 100 | 100 | 100 | 100 | 100 |
| S. SIM (3) | 0 | 100 | 0 | 100 | 0 | 100 | 100 | 0 | 100 |  | 100 | 100 | 100 | 100 | 100 | 100 | 100 |  | 0 | 0 | 0 | 0 | 0 | 0 | 0 | 0 | 0 | 0 | 100 | 100 |
